# Resonance‐Enhanced Four‐Wave Mixing Imaging for Mapping Defect Regions in Vanadium‐Doped WS_2_ Monolayers

**DOI:** 10.1002/smtd.70996

**Published:** 2026-09-03

**Authors:** Felipe Menescal, Frederico B. Sousa, Mingzu Liu, Ana P. M. Barboza, Igor F. Curvelo, Matheus J. S. Matos, Da Zhou, Bernardo R. A. Neves, Helio Chacham, Mauricio Terrones, Bruno R. Carvalho, Leandro M. Malard

**Affiliations:** ^1^ Departamento de Física Universidade Federal de Minas Gerais Belo Horizonte Minas Gerais Brazil; ^2^ Departamento de Física Universidade Federal de São Carlos São Carlos São Paulo Brazil; ^3^ Department of Physics The Pennsylvania State University Pennsylvania Pennsylvania USA; ^4^ Center for 2‐Dimensional and Layered Materials The Pennsylvania State University Pennsylvania Pennsylvania USA; ^5^ Departamento de Física Universidade Federal de Ouro Preto Ouro Preto Minas Gerais Brazil; ^6^ Department of Chemistry The Pennsylvania State University Pennsylvania Pennsylvania USA; ^7^ Department of Materials Science and Engineering The Pennsylvania State University Pennsylvania Pennsylvania USA; ^8^ Departamento de Física Universidade Federal do Rio Grande do Norte Natal Rio Grande do Norte Brazil

**Keywords:** doping, exciton, hyperspectral imaging, materials science, nanoscopic scale, optoelectronics, photoluminescence, photonics, Raman spectroscopy

## Abstract

Defect engineering is crucial for tuning 2D transition metal dichalcogenide properties for quantum and optoelectronic applications. While conventional photoluminescence (PL) and Raman spectroscopies are important characterization tools, their mapping in large area samples can be time‐consuming and lacks direct sensitivity for comprehensive defect characterization. Here, we introduce resonance‐enhanced four‐wave mixing (FWM) imaging for precise imaging and characterization of vanadium‐induced defect states in ${\mathrm{WS}}_{2}$ monolayers. Our multi‐modal investigation, integrating hyperspectral PL, Raman, and supported by density functional calculations, reveals nanoscale doping inhomogeneities, their influence on excitonic and vibrational properties. We observe resonance‐enhanced FWM signals correlating with vanadium‐induced defect regions, evidencing their unique nonlinear optical response. This work establishes FWM as an essential platform for high‐resolution, defect‐sensitive imaging, advancing defect‐engineered excitonic devices and enabling novel nonlinear quantum photonics.

## Graphical abstract

Resonant four‐wave mixing (FWM) microscopy reveals a spatial modulation of the third‐order nonlinear optical response in vanadium‐doped WS2 monolayers. This robust nonlinear contrast technique tracks the preferential accumulation of vanadium dopants along the structural bisection lines of the synthesized flakes.

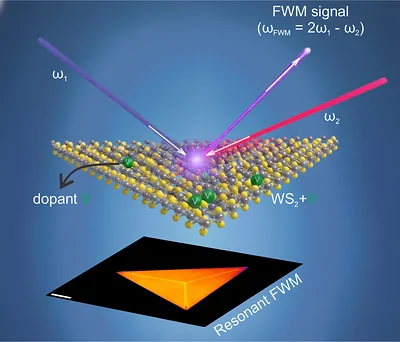

## 1 Introduction

Two‐dimensional (2D) materials hold immense promise for next‐generation quantum technologies, yet the precise spatial control and characterization of defects, which critically influence their electronic and optical behavior, remain central challenges [1, 2, 3, 4, 5]. In transition metal dichalcogenides (TMDs), the ability to engineer and probe defect states is critical for developing quantum materials, as defects modify band structures, excitonic interactions, and spin‐valley coupling [6, 7, 8, 9, 10, 11, 12, 13]. Specifically, vanadium doping in ${\mathrm{WS}}_{2}$ introduces localized electronic and magnetic states [10, 11, 14], enabling novel excitonic phenomena such as defect‐assisted transitions and altered valley polarization, highly relevant for spintronic and valleytronic applications.

While photoluminescence (PL) and Raman spectroscopy have been widely employed to study defect‐related spectral features [15], hyperspectral mapping using these linear techniques can be time‐consuming. In contrast, PL intensity imaging techniques allow rapid sample visualization, but often lack the direct sensitivity for a comprehensive understanding of defect states. This limitation is particularly pronounced in defect‐engineered systems, where severe dopant‐induced non‐radiative decay channels strongly quench spontaneous emissions. Therefore, requiring advanced nonlinear optical techniques capable of probing localized interactions with high spatial resolution and sensitivity, while offering fast acquisition times suitable for large area samples [16, 17, 18, 19, 20, 21]. Crucially, four‐wave mixing (FWM) is inherently immune to non‐radiative recombination pathways and generates a frequency‐shifted signal ($\omega_{\text{FWM}}=2\omega_{1}-\omega_{2}$) that allows zero‐background resonant collection. It is important to note that most nonlinear optical imaging methods used for defect characterization rely on second‐order processes [19], whereas works using FWM mainly focus on exciton resonances [22, 23].

Here we show that resonance‐enhanced FWM imaging provides rapid and direct insights into the nonlinear optical responses of vanadium‐induced defect states in ${\mathrm{WS}}_{2}$ monolayers. Unlike linear spectroscopies, FWM sensitively reveals subtle changes in nonlinear optical responses associated with defects states. By integrating FWM imaging with hyperspectral PL and Raman mappings, and complementing these results with density functional theory calculations, we construct a comprehensive framework for understanding the nanoscale interplay between doping, strain, and exciton states. Our findings position this nonlinear optical imaging approach, particularly FWM, as an essential technique for advanced defect characterization in 2D semiconductors, significantly enabling the development of next‐generation photonic and quantum technologies.

## 2 Results and Discussion

### 2.1 PL Mapping of Doping Inhomogeneity

Pristine and vanadium (V)‐doped ${\mathrm{WS}}_{2}$ monolayers were synthesized using a single‐step chemical vapor deposition (CVD) process, following methods reported by Zhang et al. [14] (see Methods). The pristine sample contains 0 (zero) atomic percent (at%) of vanadium (Figure 1a), while the doped samples exhibit V concentrations of approximately 0.4 at% and 2.0 at% (Figure 1b,c). This study primarily focuses on the 2.0 at% doping level, a critical regime where the ferromagnetic response is maximized [14] and defect‐induced optical phenomena are most pronounced [12, 15]. The atomic vanadium concentrations in each sample were consistent with previous characterizations reported elsewhere [14].

**FIGURE 1:**
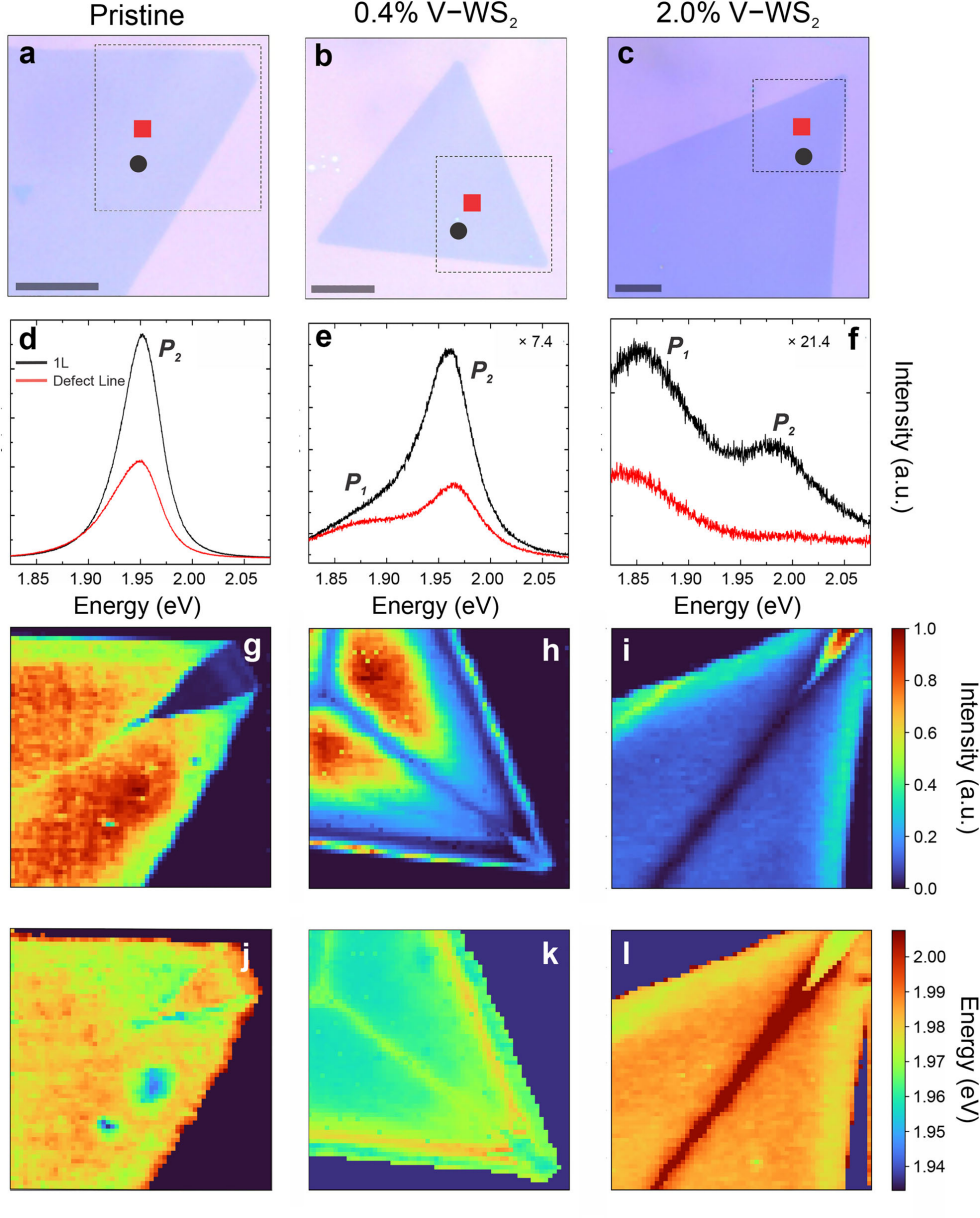
Optical imaging and PL mapping of pristine and vanadium‐doped ${\mathrm{WS}}_{2}$ monolayers. RGB true‐color white‐light reflection optical microscope images of (a) pristine, (b) 0.4 at%, and (c) 2.0 at% V‐doped ${\mathrm{WS}}_{2}$ monolayers. Scale bars represent 10 μm. Single PL spectra acquired from monolayer (1L, black curve) and defect line (red curve) regions for (d) pristine, (e) 0.4 at%, and (f) 2.0 at% V‐doped samples. PL intensity maps (integrated area) of $P_{2}$ peak for: (g) pristine, (h) 0.4 at%, and (i) 2.0 at% V‐doped. PL energy maps of $P_{2}$ peak for: (j) pristine, (k) 0.4 at%, and (l) 2.0 at% V‐doped. The spatial parameter maps were extracted from a 60×60 pixel grid over a 20×20 $\mu m^{2}$ area using the Gaussian spectral multi‐peak fitting routine detailed in Figure S1.

Figure 1d–f shows the PL spectra acquired from two distinct regions: a uniform monolayer domain (black circle) and a prominent defect line (red square), as visually marked in the optical images (Figure 1a–c). In the pristine monolayer, a single emission peak is observed at approximately 1.97 eV, characteristic of the A exciton (Figure 1d). Upon vanadium doping, this A exciton peak undergoes a notable intensity reduction, significant linewidth broadening, and energy blueshift, evolving into the $P_{2}$ peak. Meanwhile a new low‐energy peak, designated $P_{1}$, emerges only in the doped samples (Figure 1e,f). These two peaks are attributed to radiative recombination from a vanadium‐induced donor‐like state ($P_{1}$) and transitions from the conduction band minima ($P_{2}$) [11], respectively. A comprehensive discussion of their origin is detailed in prior work [11].

To spatially resolve the influence of vanadium doping on emission uniformity, we performed a hyperspectral PL mapping across 20×20 $\mu m^{2}$ region, corresponding to the dashed‐square areas in Figure 1a–c. The A exciton and $P_{2}$ intensity maps (Figure 1g–i) strikingly reveal a pronounced decrease in emission along the characteristic dark line extending from the monolayer center to its vertex. This observation is consistent with enhanced non‐radiative recombination at these structural defect sites, which act as efficient exciton sinks. Additionally, spatial variations in the localized exciton energy landscape can drive lateral exciton diffusion down the energy gradient, contributing to the localized suppression of PL intensity in those regions [24] (see Figure S2 for a direct comparison). Complementary energy maps show that while the A exciton in the pristine sample exhibits spatial uniformity (Figure 1j), the $P_{2}$ emission in doped samples displays a systematic blueshift along the defect line (Figure 1k,l) towards 2.0010±0.0032 eV (see Figure S3). In contrast, the $P_{1}$ peak, indicative of vanadium‐induced states, exhibits a clear redshift and darker contrast along this same defect line (see Figure S4), strongly suggesting preferential incorporation of vanadium dopants at these growth‐induced defect regions during synthesis [11, 24]. These PL findings highlight the significant spatial inhomogeneity introduced by vanadium doping.

Further physical characterization using scanning probe microscopy (SPM) (see Methods and Figure S5 for details) confirmed that these defect lines exhibit distinct structural, electronic, and mechanical properties. These SPM findings strongly corroborate the preferential incorporation of vanadium dopants along these growth‐induced lines, directly correlating with the observed localized PL features.

### 2.2 Raman Mapping and Strain Effects

Hyperspectral Raman measurements were performed on pristine, 0.4 at%, and 2.0 at% V‐doped ${\mathrm{WS}}_{2}$ monolayers using a 2.71 eV excitation. We focused the analysis on the same regions previously investigated with PL mapping (Figure 1). For the pristine sample, the hyperspectral frequency maps of the first‐order $E^{\prime }$ and $A_{1}^{\prime }$ modes (Figure 2a,b) reveal negligible variations in both intensity and frequency, indicating highly uniform vibrational properties. A similar homogeneous Raman response was observed for the 0.4 at% V‐doped ${\mathrm{WS}}_{2}$ monolayer (see Figure S6 for 0.4 at% V‐${\mathrm{WS}}_{2}$ shift maps and Figure S7 for intensity maps).

**FIGURE 2:**
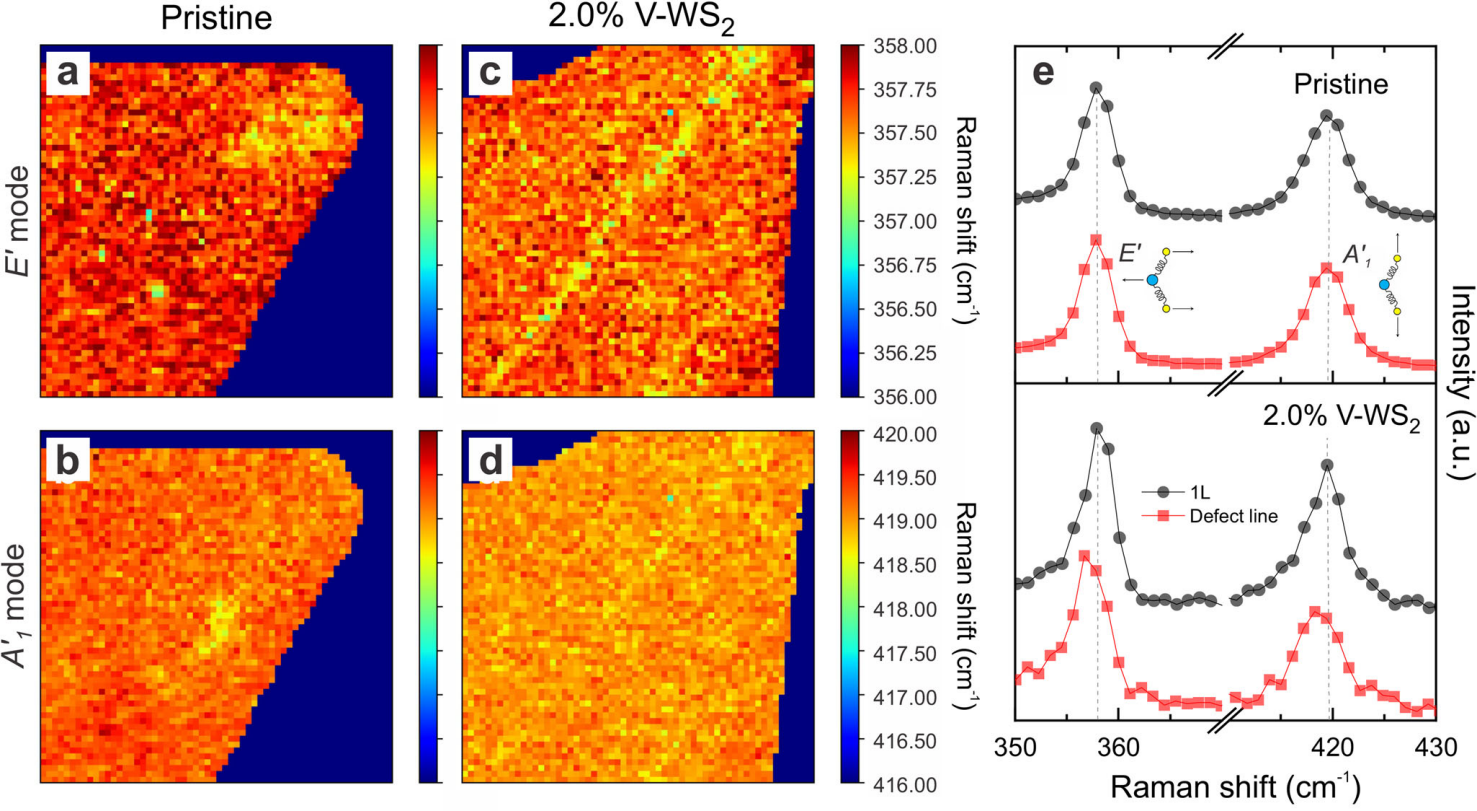
Raman hyperspectral maps of pristine and vanadium‐doped ${\mathrm{WS}}_{2}$ monolayers. (a,c) $E^{\prime }$ and (b,d) $A_{1}^{\prime }$ frequency maps for pristine and 2.0 at% V‐doped ${\mathrm{WS}}_{2}$, respectively. All maps were acquired using a 2.71 eV excitation over a 20×20 $\mu m^{2}$ area. (e) Raman spectra comparing monolayer (1L, black circles) and the defect line (red squares) regions for pristine and 2.0 at% V‐doped samples. All Raman data were normalized to the Si peak at 521.6 ${\mathrm{cm}}^{-1}$.

In stark contrast, the 2.0 at% V‐doped sample exhibits distinct spatial variations specifically along the defect line (Figure 2c,d). Here, the $E^{\prime }$ mode displays a more pronounced redshift compared to the $A_{1}^{\prime }$ mode. This behavior is mainly attributed to strain effects induced by vanadium doping, which can alter the effective mass and reduce the metal‐sulfur bond length within the ${\mathrm{WS}}_{2}$ lattice [25, 26]. Quantitatively, the observed shifts of approximately 1 ${\mathrm{cm}}^{-1}$ for $E^{\prime }$ and 0.6–0.8 ${\mathrm{cm}}^{-1}$ for $A_{1}^{\prime }$ (Figure 2e) are consistent with local strain levels ranging from 1.0–1.5% [26, 27]. Crucially, these Raman results strongly corroborate the PL findings, thereby confirming that the identified defect line corresponds to regions with enhanced vanadium doping and associated structural strain.

### 2.3 FWM Imaging of Defect States

While conventional PL and Raman spectroscopies provide valuable insights into defect distribution and local strain, hyperspectral mapping with these linear techniques can be time‐consuming, and they often lack the direct sensitivity to the nonlinear optical responses and coherent excitonic interactions crucial for a comprehensive understanding of defect states. Nonlinear optical techniques, and FWM imaging in particular, offer a distinct advantage by providing enhanced sensitivity to these subtle, localized interactions and enabling faster acquisition times [11, 18, 19, 23]. In addition, resonance‐enhanced FWM imaging, as applied in this work, provides valuable insights into their unique nonlinear optical responses. This technique selectively probes defect‐induced excitonic states by enhancing specific transitions sensitive to the local nonlinear susceptibility ($\chi^{\left(3\right)}$), thereby enabling spectroscopic access to unique defect‐signatures.

In our degenerate FWM experiment (Figure 3a), the interaction of two incident photons of tunable frequency ($\omega_{1}$) and a third photon at a fixed wavelength (1064 nm, $\omega_{2}$) generates a signal at $\omega_{\text{FWM}}=2\omega_{1}-\omega_{2}$ [23, 28, 29, 30]. Our experimental setup permits tuning of $\omega_{\text{FWM}}$ from 544 to 858 nm, thereby covering the critical ${\mathrm{WS}}_{2}$ A exciton resonance (610–680 nm). By systematically scanning $\omega_{1}$ across excitonic energies, we observe significantly enhanced FWM signals when $\omega_{\text{FWM}}$ matches real excitonic states, providing a resonance‐enhanced optical contrast (Figure 3a, inset). Although our discussion focuses on these outgoing excitonic resonances at $\omega_{\mathrm{FWM}}$, it is important to note that third‐order process can simultaneously experience incoming resonances matching the energies of the other interacting fields ($\omega_{1}$, $\omega_{2}$) or intermediate second‐order polarization pathways (such as sum‐ or difference‐frequency electronic resonances).

**FIGURE 3:**
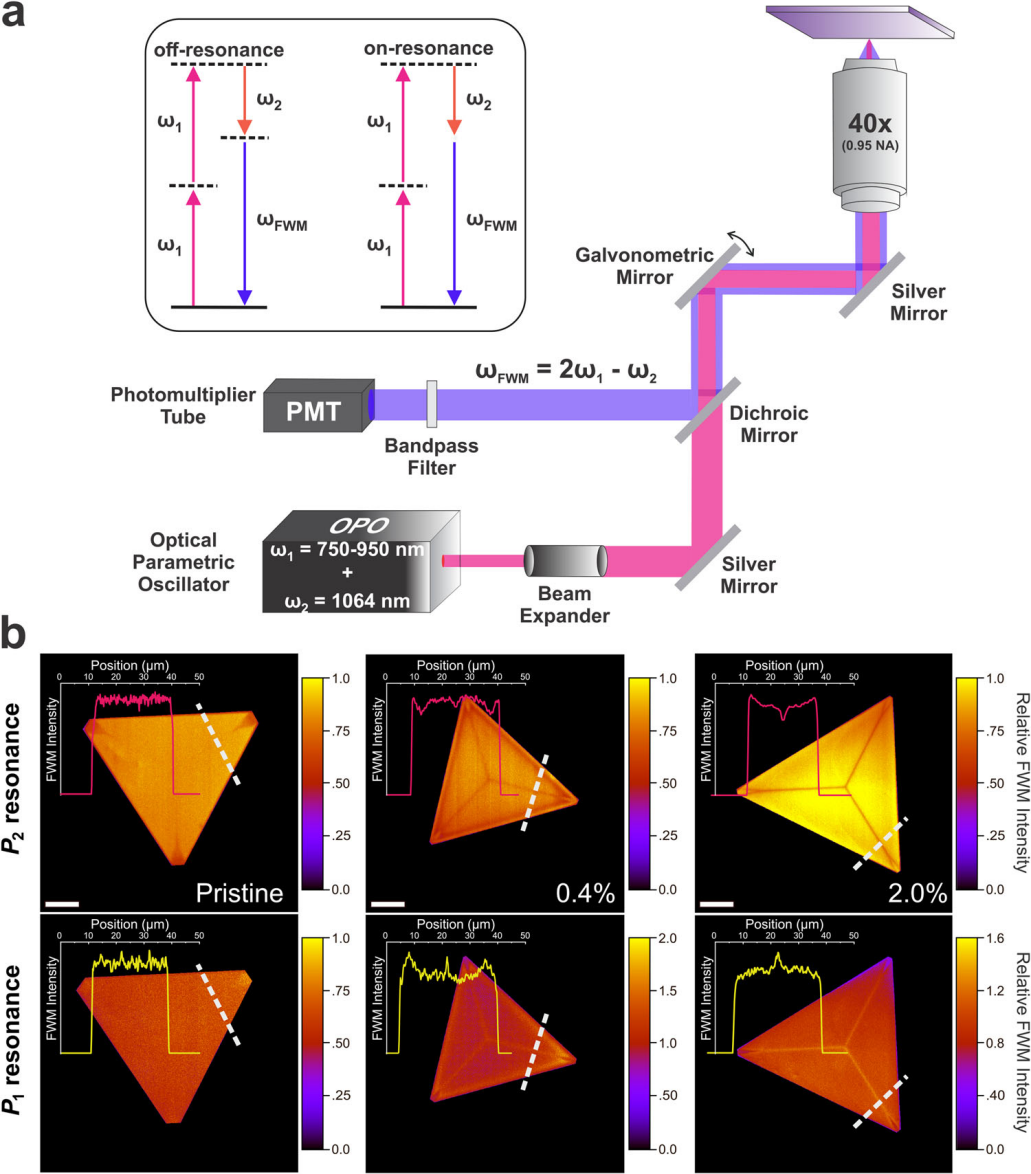
FWM imaging of pristine and vanadium‐doped ${\mathrm{WS}}_{2}$ monolayers. (a) Schematic of the FWM experimental setup. Further details are in the Methods section. The inset shows energy diagrams of the FWM generation process, illustrating off‐resonance (left) and on‐resonance (right) conditions. (b) FWM images of pristine, 0.4 at%, and 2.0 at% V‐doped ${\mathrm{WS}}_{2}$ monolayers with FWM signal at 2.03 eV $P_{2}$ free‐exciton resonance, top row) and 1.82 eV ($P_{1}$ defect‐bound resonance, bottom row), respectively. Scale bars are 20 μm.

Figure 3b shows the acquired FWM images of pristine, 0.4 at%, and 2.0 at% V‐doped ${\mathrm{WS}}_{2}$ samples at two distinct FWM signal energies: 2.03 eV (610 nm, off‐resonance) and 1.82 eV (680 nm, on‐resonance with the $P_{1}$ defect‐related peak). Notably, in the doped samples, the FWM response is strongly modulated along the previously identified defective lines by resonant excitation. At the $P_{2}$ resonant energy (2.03 eV, top row), both 0.4 at% and 2.0 at% V‐doped samples exhibit a distinct FWM quenching along the defective lines, manifesting as a dark contrast. The 0.4 at% sample additionally shows dark contrast near flake edges. Conversely, when the FWM signal is tuned to the $P_{1}$ resonant energy of 1.82 eV (bottom row), we observe an enhancement of FWM emission along these same regions, appearing with bright contrast.

The FWM images clearly delineate two spatially distinct regions: (i) the one‐dimensional bisection lines (from center to vertices), which our prior PL and SPM results confirmed as preferential sites for dopant incorporation, and (ii) the surrounding monolayer flake area. The observed reduction in FWM intensity along defect lines at 2.03 eV, which contrasts sharply with the stronger FWM signals in these same defect regions at 1.82 eV of V‐doped samples, provides compelling direct evidence of increased vanadium‐induced defect states. These observations demonstrate that vanadium‐induced defect states strongly modulate nonlinear excitonic interactions, most likely by significantly enhancing third‐order nonlinear susceptibility $\chi^{\left(3\right)}$, localized at these defect sites.

To evaluate the resonant modulation of the nonlinear optical response along the defect lines, we extracted spatial intensity profiles across the flakes (indicated by the dashed white lines in Figure 3b). The localized FWM intensities from the defect line ($I_{\mathrm{DL}}$) were normalized to the adjacent monolayer background ($I_{1L}$), where $I_{1L}$ represents a uniform baseline averaged along the defect‐free regions of the same line profile. Following a direct comparison approach, we define the spectral contrast factor (C) at the defect line as:

$$C=\frac{I_{\mathrm{DL}}\left(P_{1}\right)-I_{\mathrm{DL}}\left(P_{2}\right)}{I_{\mathrm{DL}}\left(P_{1}\right)+I_{\mathrm{DL}}\left(P_{2}\right)},$$

where $I_{\mathrm{DL}}\left(P_{1}\right)$ and $I_{\mathrm{DL}}\left(P_{2}\right)$ correspond to the normalized defect‐line intensities under $P_{1}$ defect‐bound resonance (1.82 eV) and $P_{2}$ free‐exciton resonance (2.03 eV) excitation conditions, respectively. Quantitative analysis of these traces reveals a well‐defined contrast factor of C=0.14±0.02 for the 0.4 at% V‐doped sample, indicating a clear resonant‐driven brightness modulation at the defect region. Similarly, the 2.0 at% doped sample exhibits a contrast factor of C=0.16±0.02. This comparable contrast, despite the significantly higher overall dopant density in the 2.0 at% sample, can be attributed to the heavily doped bisection lines approaching optical and structural saturation.

To further investigate the spectral characteristics of these FWM resonances, Figure 4 shows the FWM intensity as a function of the generated photon energy (ranging from 1.82–2.03 eV) for pristine, 0.4 at%, and 2.0 at% doped ${\mathrm{WS}}_{2}$ samples. Data was acquired from both the monolayer (blue circles) and defect line (red squares) regions (see Figures S8–S10 for corresponding resonant FWM images). In the pristine sample, the FWM response exhibits a single optical transition at approximately 1.95 eV in both regions. This feature is attributed to a resonance near the A exciton energy, where the emitted $\omega_{\text{FWM}}$ is in resonance. This same characteristic feature persists in both the 0.4 at% and 2.0 at% doped samples but shows a systematic redshift to approximately 2.0 eV, strongly correlating with the $P_{2}$ peak energy previously observed in our PL measurements. Significantly, in the V‐doped samples, excitations between 1.85 and 1.95 eV reveal an additional, distinct resonance near 1.88 eV in both monolayer and defect line regions. It is worth to note that probing energies below 1.85 eV was limited by our optical setup.

**FIGURE 4:**
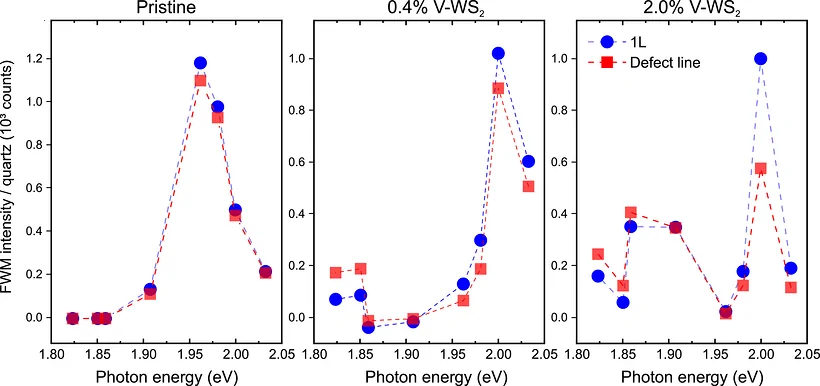
Resonance‐enhanced FWM response of pristine and vanadium‐doped ${\mathrm{WS}}_{2}$ monolayers. FWM excitation profiles for pristine, 0.4 at%, and 2.0 at% doped ${\mathrm{WS}}_{2}$ samples acquired from the monolayer (red squares) and defect line (blue circles) regions. Intensities are normalized by the quartz reference signal at each excitation energy.

Overall, these results demonstrate the ability of resonance‐enhanced FWM imaging to detect and characterize defect‐related optical states in V‐doped ${\mathrm{WS}}_{2}$ monolayers. The unique resonance‐enhanced signal offers direct insights into defect‐induced excitonic transitions, highlighting FWM's high spatial sensitivity and capability to reveal local modifications in the nonlinear optical response. In our FWM images, the experimental spatial resolution is close to 500 nm by using a 0.95 NA objective (see Methods), and the acquisition times ranges from a few to 30 seconds. Therefore, such characteristics reinforces FWM's imaging as potential tool for rapid and high spatial resolution for advanced defect characterization in doped 2D semiconductors.

We note that while the $P_{2}$ resonance exhibits a slight apparent narrowing in our FWM spectra at higher doping concentrations, this feature is attributed to the coarse spectral sampling step of our pump‐wavelength tuning. When resolved with higher spectral resolution, as demonstrated by Sousa et al. [11], the $P_{2}$ resonance undergoes the expected physical broadening due to dopant‐induced disorder and scattering. The comparable contrast observed between our 0.4 at% and 2.0 at% samples further suggests that the heavily doped bisection lines are approaching structural and optical saturation, which restricts further absolute changes in the differential non‐linear susceptibility ($\chi^{\left(3\right)}$).

The physical origin of this contrast behavior tracks the distinct electronic transitions involved under different excitation energies. As supported by our linear PL data, with increasing vanadium concentration, the PL intensities of both $P_{1}$ and $P_{2}$ decrease, while the $P_{1}/P_{2}$ intensity ratio progressively increases. This trend indicates that the $P_{1}$ transition becomes increasingly dominant relative to $P_{2}$ upon V incorporation. Such behavior can be attributed to the enhanced density of available states associated with the vanadium‐induced defect band, accompanied by the progressive perturbation and suppression of the free‐exciton transition.

In our resonant FWM imaging, this state‐population shift is directly observed in the variation of the contrast between the monolayer and the defect line for the doped ${\mathrm{WS}}_{2}$ monolayers. A lower vanadium concentration (monolayer domain) results in a stronger optical transition at the $P_{2}$ resonance, while higher vanadium doping (defect lines) leads to a relatively larger optical response at the $P_{1}$ resonance. Because the overall FWM intensity is a coherent process immune to the additional non‐radiative decay channels that quench linear PL, this dopant‐dependent effect is straightforwardly probed through the inverse FWM contrast at the $P_{1}$ and $P_{2}$ resonances (see Section S7). Following these considerations, the resilience of the nonlinear signal along the heavily perturbed defect boundaries can be further tied to localized electronic configurations and modified carrier population lifetimes within the defect band (see Figures S11 and S12). A similar phenomenon of robust, resonance‐enhanced FWM at localized defect levels has been documented in other low‐dimensional systems, highlighting how localized electronic states can drive prominent parametric responses independently of linear emission efficiency [21].

### 2.4 Density Functional Theory Calculations

To elucidate the mechanisms underlying the observed optical phenomena, specifically the A exciton quenching, energy shift, and FWM response, we performed Density Functional Theory calculations within the DFT+U framework (see Methods). We first examine the thermodynamic tendency for V incorporation, local enrichment, and substitutional disorder at different concentrations. We then analyze how a single V substitution and increasing V concentration perturb the electronic structure and optical response.

To examine the impact of higher doping concentrations and the inherent disorder in these systems, we employed the Special Quasirandom Structures (SQS) method [31, 32, 33] to model various vanadium concentrations (x=0.04to0.32) within a 5×5 supercell. The formation energies, $E_{f}$, of the SQS structures were determined using the equation:

(1) $$E_{f}=\frac{E_{W_{1-x}V_{x}S_{2}}-\left(1-x\right)E_{{\text{WS}}_{2}}-xE_{{\text{VS}}_{2}}}{\text{NV}},$$

where $E_{W_{1-x}V_{x}S_{2}}$ is the total energy of the disordered alloy supercell, $E_{{\mathrm{WS}}_{2}}$ and $E_{{\mathrm{VS}}_{2}}$ are the total energies of pristine ${\mathrm{WS}}_{2}$ and ${\mathrm{VS}}_{2}$ calculated for equivalent supercells, and NV is the number of V atoms. This quantity corresponds to the zero‐temperature mixing energy of $W_{1-x}V_{x}S_{2}$ relative to the segregated ${\mathrm{WS}}_{2}$ and ${\mathrm{VS}}_{2}$ end‐member phases, normalized by the number of incorporated V atoms. The calculated energies are positive over the investigated concentration range, indicating that the homogeneous alloy lies above the tie line between the segregated end‐member phases at 0 K. However, the average energetic cost per V atom decreases from 358.8 to 216.3 meV as x increases from 0.04 to 0.32, suggesting non‐ideal alloying and an effective tendency toward V‐rich configurations.

At finite temperature, the configurational entropy contributes to the mixing free energy according to $\Delta F_{\mathrm{mix}}\left(x,T\right)=\Delta H_{\mathrm{mix}}\left(x\right)-T\Delta S_{\mathrm{mix}}$, with $\Delta S_{\mathrm{mix}}=-k_{B}\left[x\ln x+\left(1-x\right)\ln\left(1-x\right)\right]$ per metal site [34]. At the CVD growth temperature, this entropic contribution can partially compensate the positive mixing enthalpy and make V incorporation thermodynamically more accessible. Following the concentration‐dependent chemical‐potential analysis of Wang et al. [34] for V‐doped ${\mathrm{WS}}_{2}$, we further evaluated the thermodynamic behavior of the $V_{x}W_{1-x}S_{2}$ alloy using our SQS formation energies (see Figure S14). When the pure ${\mathrm{VS}}_{2}$ end‐member limit is included, the resulting critical concentration is $x_{c}\approx 0.20$, in close agreement with the value reported by Wang et al. This behavior supports a tendency toward V enrichment and concentration phase separation, consistent with the experimentally observed non‐uniform dopant distribution, although local enrichment at defect lines may also be influenced by growth kinetics and defect‐site energetics [35].

Having established the relevance of V enrichment and substitutional disorder, we next analyze the electronic‐structure perturbation induced by an isolated V substitution. A single tungsten (W) atom was replaced by a vanadium (V) atom in a 7 × 7 supercell, corresponding to a V concentration of approximately 2%. As shown in Figure 5a, the calculated unfolded band structure reveals a redistribution of primitive‐cell spectral weight around the **K**/**K** 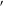 valleys upon V substitution. This redistribution reflects the perturbation of the band‐edge states by the V defect. Because localized defect‐related states may carry weak or diffuse primitive‐cell spectral weight in the unfolded representation, we additionally show the folded and atom‐projected band structures in Figures 5b and S15. These complementary projections identify V‐related states within or near the pristine gap and clarify their hybridization with the band‐edge states.

**FIGURE 5:**
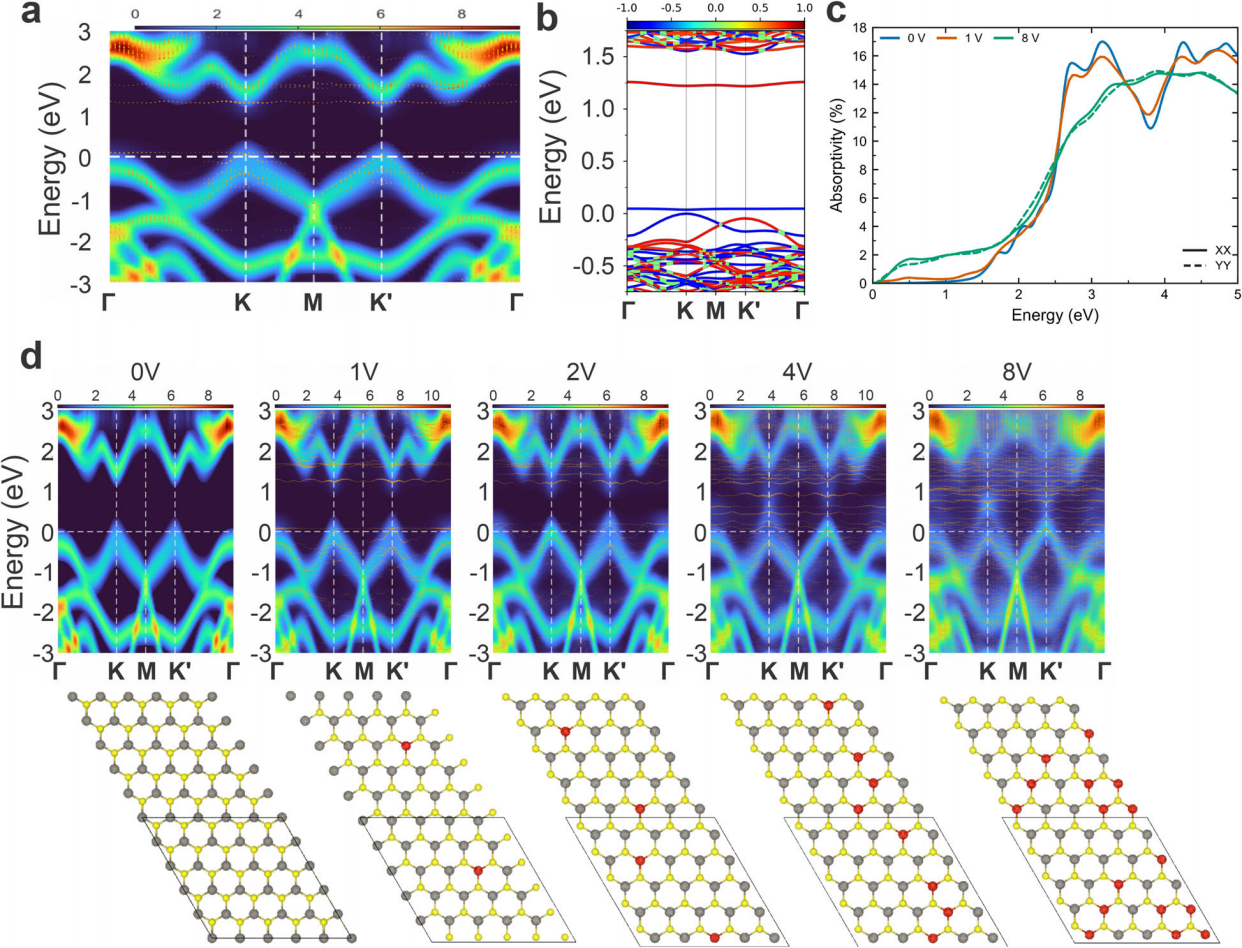
Density functional theory calculations of vanadium‐doped ${\mathrm{WS}}_{2}$ monolayers. (a) Unfolded band structure of a 7×7 ${\mathrm{WS}}_{2}$ supercell with one substitutional V atom. The color scale represents the primitive‐cell spectral weight, while the circles indicate the projection onto V‐derived states. (b) Folded supercell band structure projected onto the out‐of‐plane spin components, $S_{z+}$ (red) and $S_{z-}$ (blue). (c) Calculated 2D absorptivity spectra for pristine, one‐V, and eight‐V ${\mathrm{WS}}_{2}$. Solid and dashed lines correspond to XX and YY light polarizations, respectively. (d) Evolution of the unfolded band structures (top) and corresponding 5 × 5 supercell atomic structures (bottom) for $N_{V}=$ 0, 1, 2, 4, and 8 (see Figure S13).

The in‐gap/near‐gap states are primarily associated with $d_{z^{2}}$ orbitals of V and neighboring W atoms (see Figures S16–S21), consistent with previous reports [11, 12, 15]. We also observe a breaking of valley symmetry between the **K** and **K** 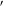 points [12, 15]. Notably, the introduction of V also leads to a reduction of Bloch character in the calculated effective band structure, particularly near **K** valleys. The reduced primitive‐cell Bloch spectral weight near the K valleys reflects hybridization between V‐induced localized states and the band‐edge states. This defect‐induced mixing perturbs the extended valley states involved in bright excitonic recombination, providing a qualitative microscopic picture of how V‐related defects can promote defect‐ assisted relaxation and contribute to the observed PL quenching [36, 37, 38, 39].

Figure 5b shows the folded supercell band structure projected onto the out‐of‐plane spin components $S_{z+}$ and $S_{z-}$, resolving the spin character of the V‐induced and band‐edge states near **K**/**K** 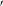. Since spin and valley degrees of freedom are coupled in monolayer TMDs, this spin‐resolved representation is important for identifying how magnetic V substitution perturbs valley‐selective states. Similar spin‐resolved DFT+U analysis in V‐doped ${\mathrm{WSe}}_{2}$ showed that V‐induced magnetic moments split the valence‐band edges at **K** and **K** 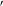, consistent with the observed giant valley Zeeman response [12]. Thus, the present ${\mathrm{WS}}_{2}$ results support a related qualitative picture in which V‐induced spin‐polarized defect/band‐edge states modify the valley‐resolved electronic structure and contribute to defect‐related optical channels.

Figures 5d and S13 show the evolution of the electronic structure of the system with increasing vanadium concentration. The unfolded band structures show a well‐defined Bloch character for the valence band maximum and conduction band minima of pristine ${\mathrm{WS}}_{2}$. At low concentrations (e.g., 1 and 2  V), a new band emerges below the pristine conduction band, characterized by states with limited Bloch character and visibility only at the K and $K^{\prime }$ points. At higher concentrations (e.g., 4V atoms or more), a significant introduction of additional defect states occurs within the mid‐gap region. Importantly, these mid‐gap states become $K-K^{\prime }$ asymmetric, and the overall system begins to exhibit characteristics of a transition toward metallic behavior, with a non‐zero spectral density across the entire pristine bandgap. These electronic‐structure changes provide a microscopic basis for the observed A‐exciton energy shift and PL quenching, as V‐induced states redistribute the primitive‐cell spectral weight near the band‐edge region.

Because the microscopic assignment of the $P_{1}$ and $P_{2}$ resonances depends on the Hubbard correction applied to the V d orbitals, as discussed by Sousa et al. [11], we also compared representative one‐V‐doped ${\mathrm{WS}}_{2}$ calculations using U= 2.5 and 3.0 eV (see Figure S21). This comparison shows that the qualitative electronic‐ structure features relevant to the present work are unchanged: V substitution introduces defect‐related states within or near the pristine gap and perturbs the band‐edge states around **K**/**K** 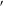. Accordingly, the DFT+U results are used here to support the presence and orbital character of V‐induced defect/band‐edge hybridized states, while the detailed microscopic assignment of $P_{1}$ and $P_{2}$ remains model‐dependent.

The optical absorption spectra (Figure 5c) provide a useful connection to the FWM measurements. Pristine ${\mathrm{WS}}_{2}$ exhibits well‐defined first and second optical transitions at 1.72 and 2.07 eV, with maximum absorption at 3.1 eV. Upon vanadium incorporation, the onset of absorption shifts to the infrared (≈0.5 eV), indicating that the V‐induced mid‐gap states are optically active. The increase of this low‐energy spectral weight with V concentration is consistent with the resonance‐enhanced FWM response observed in defect‐rich regions, where nonlinear spectroscopy is sensitive to transitions involving defect‐derived electronic states even when radiative PL is strongly quenched. Because these spectra were calculated at the independent‐particle DFT level and do not explicitly include electron‐hole interactions, the comparison with the experimental optical response should be understood at a semiquantitative level. Taken together, our theoretical analysis addresses two complementary aspects: the electronic‐structure perturbation induced by a single V substitution and the evolution of defect‐derived states with increasing dopant concentration and disorder.

## 3 Conclusion

We have applied and demonstrated a resonance‐enhanced FWM imaging methodology to characterize defect states in vanadium‐doped ${\mathrm{WS}}_{2}$ monolayers. Our multi‐modal spectroscopic approach, which includes hyperspectral PL and Raman mapping, unveiled new excitonic features and spectral shifts indicative of nanoscale doping inhomogeneity. DFT calculations supported these findings by confirming that vanadium substitution introduces optically active mid‐gap states, which reduce the Bloch character and explain the observed PL quenching and blueshift. The FWM technique emerges as a powerful, rapid, and spatially sensitive alternative to linear spectroscopies, as it selectively probes these theoretically confirmed defect states. This work establishes FWM as a pivotal tool for advanced defect engineering in 2D semiconductors, holding immense promise for next‐generation quantum photonics and optoelectronic devices.

## 4 Methods

### 4.1 Sample Preparation

The ${\mathrm{WS}}_{2}$ monolayers, both pristine and vanadium‐doped, were fabricated using a single‐step chemical vapor deposition (CVD) process. The sample exhibited regular triangular shapes. By carefully controlling the concentrations of vanadium precursors during synthesis, flakes with average vanadium concentrations of 0.4 at% and 2.0 at% were obtained. The average nominal vanadium concentrations of approximately 0.4 at% and 2.0 at% correspond to calibrated CVD growth batches verified via high‐angle annular dark‐field scanning transmission electron microscopy (HAADF‐STEM) counting statistics [14]. It is important to note, however, that the local dopant density can deviate spatially across an individual flake. Specifically, recent synchrotron‐based X‐ray fluorescence (XRF) microscopy on similarly processed flakes reveals a distinct chemical enrichment of vanadium localized along the structural defect boundaries [35]. Therefore, while the nominal percentages accurately define the low‐ and moderate‐doping regimes of the overall domain, the structural lines act as regions of elevated local dopant density, perfectly corroborating the distinct, localized electronic signatures observed in our optical mappings.

### 4.2 Raman and PL Measurements

Raman and PL spectra were acquired using a WITec Alpha 300 micro‐Raman spectrometer. For Raman measurements, 457 nm excitations laser at ∼300 μW power and an 1800 lines/mm grating were employed with resolution better than 1 ${\mathrm{cm}}^{-1}$. PL measurements utilized a 532 nm excitation laser at ∼150 μW power with a 600 lines/mm grating. Data collection was performed using an electron multiplying charge‐coupled device (EMCCD). A Zeiss 100× objective (numerical aperture = 0.9) was used for both laser focusing and signal collection. All measurements were conducted at room temperature at the LCPNano facilities at UFMG. Spectral analysis was performed using PortoFlow (FabNS) software. Raman spectra were fitted with Lorentzian functions for the $E^{\prime }$ and $A_{1}^{\prime }$ modes, and for the silicon substrate reference peak. The intensity of each spectrum was normalized based on the silicon peak's intensity and position. PL spectra were fitted using Gaussian function for the $P_{1}$ and $P_{2}$ excitonic peaks (Figure S1).

### 4.3 Fluorescence Imaging

Optical microscopy images (Figure S22) were collected using an Nikon Eclipse upright microscope coupled with a Nikon fs‐fi02 camera. A 60× objective lens with a numerical aperture (NA) of 0.85 was used for image acquisition. An Hg lamp as an exciting light source. This light is then directed through the objective and onto the specimen by a special dichroic mirror that reflects certain wavelengths while passing through others (green long pass).

### 4.4 Nonlinear Optical Measurements

FWM experiments were conducted using an APE Berlin picoEmerald optical parametric oscillator (OPO) beam system. This system provides a tunable output ($\omega_{1}$) in the range of 750–950 nm (1.35–1.65 eV) and a fixed output ($\omega_{2}$) at 1064 nm (1.16 eV). The OPO operated with a 7 ps pulse width and an 80 MHz repetition rate. Backscattered nonlinear FWM signals were collected by the same objective used for excitation and directed to a photomultiplier tube (PMT) via dichroic mirror. The total incident laser power was kept at ∼70 mW at the sample, equally distributed between the two beams across all measurements. FWM signals were generated at $\omega_{\text{FMW}}=2\omega_{1}-\omega_{2}$, and by tuning $\omega_{1}$ in the 775–830 nm (1.49–1.60 eV) range, FWM emissions within 610–680 nm (1.82–2.03 eV) were detected, covering the ${\mathrm{WS}}_{2}$ A exciton resonance. A 40× objective (numerical aperture = 0.95) was used for beam focusing and signal collection. A specific set of optical filters was employed to select the desired FWM wavelength, as detailed in Table S1 of the Supporting Information. To eliminate incoherent background contributions, spatial FWM maps were recorded both at zero time delay and at a 25 ps temporal delay between the excitation pulses, with the latter subtracted to isolate the instantaneous third‐order nonlinear signal (see Figure S23). Second Harmonic Generation (SHG) imaging, presented in Figure S24, at 405 and 395 nm, was performed using the identical optical setup by collecting the SHG signal instead of FWM. For FWM intensity profile generation (Figure 4), raw counts (intensity) from each FWM image at a specific wavelength were processed by subtracting the background response from the silicon substrate and normalizing by counts from a quartz reference standard. Consistency was ensured by maintaining the same laser wavelengths, incident power, and filter configurations throughout all FWM measurements. This analysis was extended to other flakes to ensure the same phenomena was observed (see Figure S25). A schematic of the nonlinear optical experiment setup is shown in Figure 3.

### 4.5 Statistical Analysis of Experimental Data

#### 4.5.1 FWM Image Normalization

The spatial FWM intensity maps represented in Figure 3b evaluate three distinct monolayer flakes across different vanadium dopant concentrations. To ensure quantitative consistency across different imaging conditions, the intensity scale of each map was normalized by its respective local monolayer background. For each image, the reference background intensity ($I_{1L}$) was determined by calculating the mean pixel intensity extracted from a representative rectangular area (ranging from 10 × 10 to 15 × 15 square micrometers) located entirely within the pristine 1L region.

#### 4.5.2 FWM Resolution Determination

Spatial resolution of FWM microscope configuration was quantified via the knife‐edge method across a sharp structural boundary. A linear intensity profile was extracted across the boundary interface within the flake limits, and its first spatial derivative was calculated. The resulting derivative profile was fitted using a Gaussian amplitude function, where the standard deviation parameter w is directly related to the Full Width at Half Maximum, defined as $\mathrm{FWHM}=2\sqrt{2\ln2}w$. The spatial resolution of the FWM setup was experimentally determined to be 0.528 ± 0.037 (≈ 528 nm) via a Gaussian fit of the first derivative of an edge intensity profile, as detailed in Section S14, Figure S26 of the Supporting Information.

#### 4.5.3 Resonant FWM Profile

To extract the pure resonant profiles and eliminate slower incoherent background contributions, spatial FWM maps were collected under concurrent pulse arrival (0 ps delay) and a temporal delay of 25 ps, with the latter systematically subtracted. The substrate background contribution from the underlying silicon was subsequently removed. To ensure robust statistical sampling, cross‐sectional line profiles were extracted using an integrated linewidth of 8 to 10 μm. The mean intensity values and their associated standard deviations were evaluated at both the local coordinate centers (the defect lines) and the outer spatial zones (the pristine monolayer regions) to construct the quantitative resonant profiles displayed in Figure 4 (and the representative examples in Section 13, Figure S25 of the Supporting Information).

#### 4.5.4 Data Presentation and Uncertainty

Unless specified otherwise, all quantitative data points, peak positions, and contrast values are presented as the calculated mean value ± standard deviation. Experimental error bars depicted in all spectroscopic plots and spatial profiles reflect these standard deviations, evaluated across multiple independent measurements (n≥3) on different sample regions to ensure reproducibility. Linear and non‐linear curve fitting procedures, including peak‐shape analyses, with the reported parameter uncertainties corresponding directly to the standard errors derived from the fit covariance matrix.

#### 4.5.5 Theoretical Calculation

All calculations were performed using the Vienna Ab initio Simulation Package (VASP) code, employing the projector‐augmented wave (PAW) method [40, 41, 42, 43] within the density functional theory with on‐site Coulomb correlations (DFT+U) formalism [44, 45, 46]. The exchange‐correlation functional was described by the Perdew‐Burke‐Ernzerhof (PBE) parametrization [47]. On‐site Coulomb correlations were accounted for via $U_{eff}=3.0$ eV applied to the d−orbitals in vanadium atoms [48, 49]. Spin‐orbit coupling (SOC) was included in all calculations [50]. A plane‐wave basis set with an energy cutoff of 400 eV was used, and the energy convergence criterion was set to $10^{-6}$ eV. Periodic boundary conditions were applied with a minimum separation of 13 Å along the z‐direction to eliminate interlayer interactions. The reciprocal space grid was constructed using Γ‐centered meshes: 8×8×1 for the 3×3 supercell, 6×6×1 for the 5×5 supercell, and a 3×3×1 for the 7×7 supercell. To characterize the random alloy $W_{1-x}V_{x}S_{2}$, the SQS method, implemented in the ICET software [31, 32, 33], as employed. This method facilitates the accurate estimation of intrinsic properties of random alloys, while maintaining their physical characteristics, even at finite scales. A 5×5 supercell (25 tungsten sites) was constructed to model target vanadium concentrations ranging from 4% to 32%. For each concentration, the most representative structural configuration was selected for further calculations. The SQS structures were generated using cutoff radii of 13.5, 6.0, and 4.0 Å to optimize the correlation functions of first, second, and third nearest neighbors, respectively. Band structure analyses and transition dipole moment calculations utilized the pyband and VASPKIT software packages [39, 51].

#### 4.5.6 Scanning Probe Microscopy Measurements

Atomic force microscopy (AFM) and lateral force microscopy (LFM) measurements were performed using a CSC37 No Al probe (MikroMash, with a nominal spring constant k=0.3 N/m and resonant frequency $\omega_{0}=20$ kHz). Electrostatic force microscopy (EFM) experiments were conducted with a HQ:XSC11 probe (MikroMash, k=7 N/m and $\omega_{0}=155$ kHz). All scanning probe microscopy experiments were carried out using a Nanoscope V MultiMode8 SPM (Bruker Instruments).

## Author Contributions

F.M., F.B.S., and L.M.M. conceived the study. F.M. and F.B.S. performed Raman, PL, FWM, and SHG measurements. A.P.M.B. performed and B.R.A.N. analyzed SPM experiments. M.L., and M.T. prepared the samples. F.M., F.B.S., B.R.C., and L.M.M. analyzed the optical data. M.J.S.M. and I.F.C. performed DFT calculations. F.M., F.B.S, B.R.C., M.J.S.M., H.C., and L.M.M. interpreted the results and wrote the manuscript. B.R.C., and L.M.M. supervised the project. All authors reviewed and agreed on the final version of the manuscript.

## Conflicts of Interest

The authors declare no conflicts of interest.

## Supporting information


**Supporting File**: smtd70996‐sup‐0001‐SuppMat.pdf.

## Data Availability

The data that support the findings of this study are available from the corresponding author upon reasonable request.
