# Supplementary material for: Resonance‐Enhanced Four‐Wave Mixing Imaging for Mapping Defect Regions in Vanadium‐Doped WS2 Monolayers
Source: Small Methods. 2026 Sep 3;10(19):e70996. doi: 10.1002/smtd.70996 (PMC13646075; doi:10.1002/smtd.70996)
Supplement: Supplementary file 1 [file SMTD-10-e70996-s001.pdf]

Supporting Information for

Resonance-Enhanced Four-Wave Mixing Imaging for Mapping Defect Regions in Vanadium-Doped WS_2 Monolayers

Felipe Menescal, Frederico B. Sousa, Mingzu Liu, Ana P. M. Barboza, Igor F. Curvelo, Matheus J. S. Matos, Da Zhou, Bernardo R. A. Neves, Helio Chacham, Mauricio Terrones, Bruno R. Carvalho*, Leandro M. Malard*

*Correspondence to: brunorc@fisica.ufrn.br, lmalard@fisica.ufmg.br

Section 1 Photoluminescence (PL) mapping details: intensity features, peak-fitting, and P_1 peak for V-doped samples

For the fitting routine, the pristine WS_2 spectra was successfully reconstructed using a single Gaussian profile corresponding to the intrinsic free-exciton emission (P_2). For both the 0.4 at% and 2.0 at% V-doped samples, a two-peak Gaussian model was implemented to decouple the overlapping contributions of the P_1 (defect-bound) and P_2 (free-exciton) states. The extracted peak intensities were subsequently normalized by the maximum peak intensity of each spectrum to map the spatial variations. The small and featureless residuals confirm the mathematical validity and robustness of our spectral decomposition.

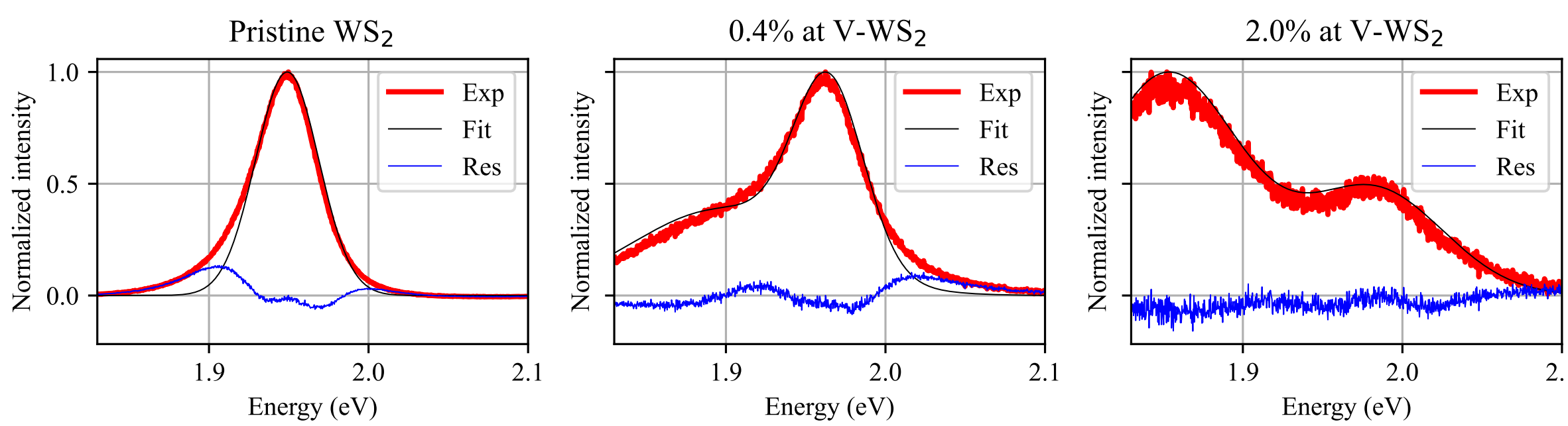

Supplementary Figure S1: **PL fitting examples.** PL fit examples. The peaks were fitted with Gaussian, then normalized by the most intense peak. Two peaks were used for both 0.4 and 2.0 at% V-WS2, but only one was used for the pristine sample.

To allow a direct comparison of emission strengths, all PL spectra were recorded under identical laser power and acquisition times, with absolute intensities displayed in common axis to demonstrate the strong dopant-induced PL quenching.

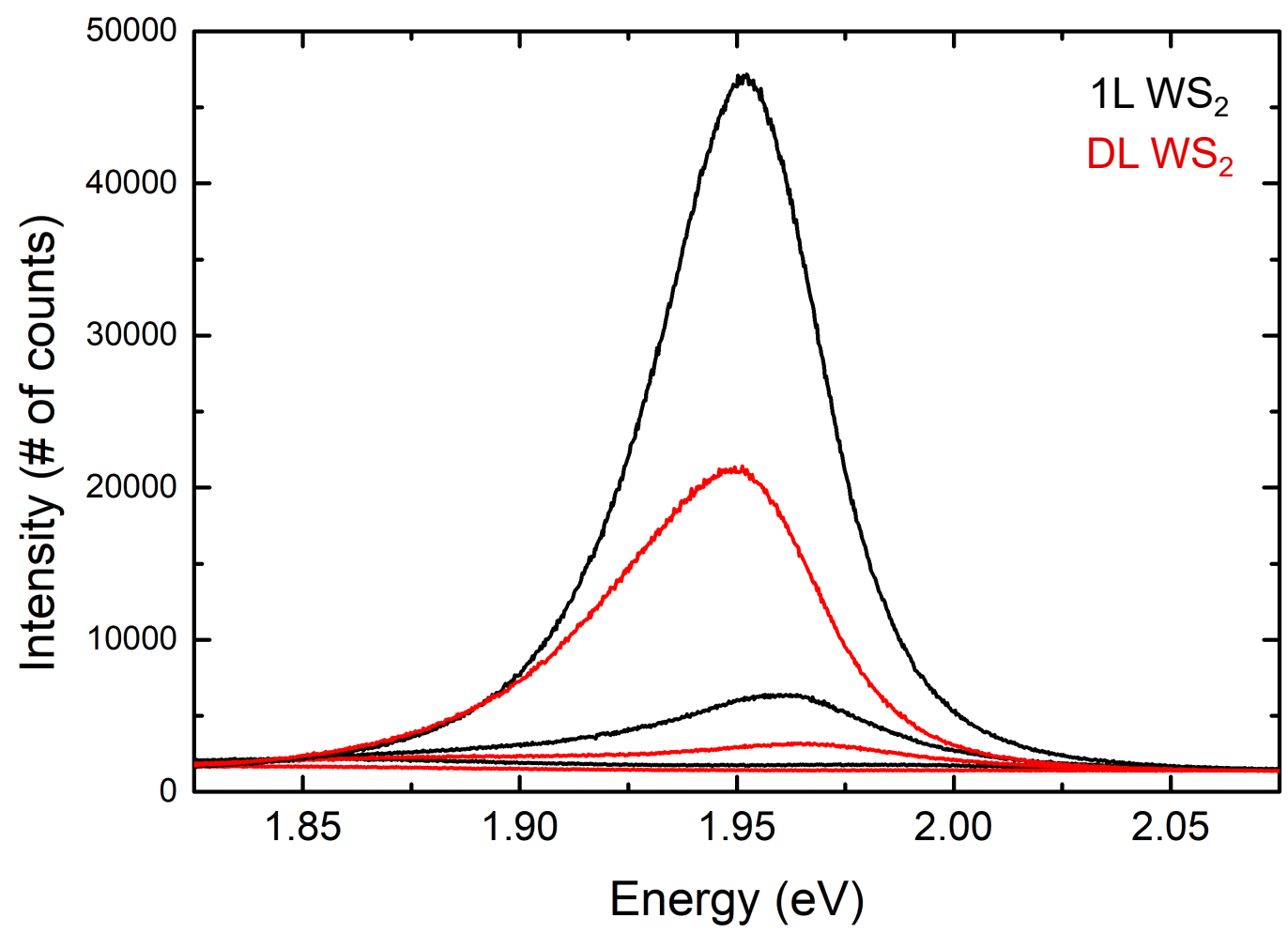

Supplementary Figure S2: **Absolute PL spectra.** The shown spectra are the same show in Figure 1. The black lines are the response of the monolayer Pristine, 0.4, and 2.0% at V-WS_2, respectively in decreasing order, same for the red lines that represent the defect line response. The spectra were acquired using the same laser power and acquisition times, allowing thus a direct comparison between PL intensities. Monolayer pristine presents a absolute intensity of 47k counts, while 0.4% presents 6.4k, and 2.0% only 2.2k for 1 second acquisition spectrum.

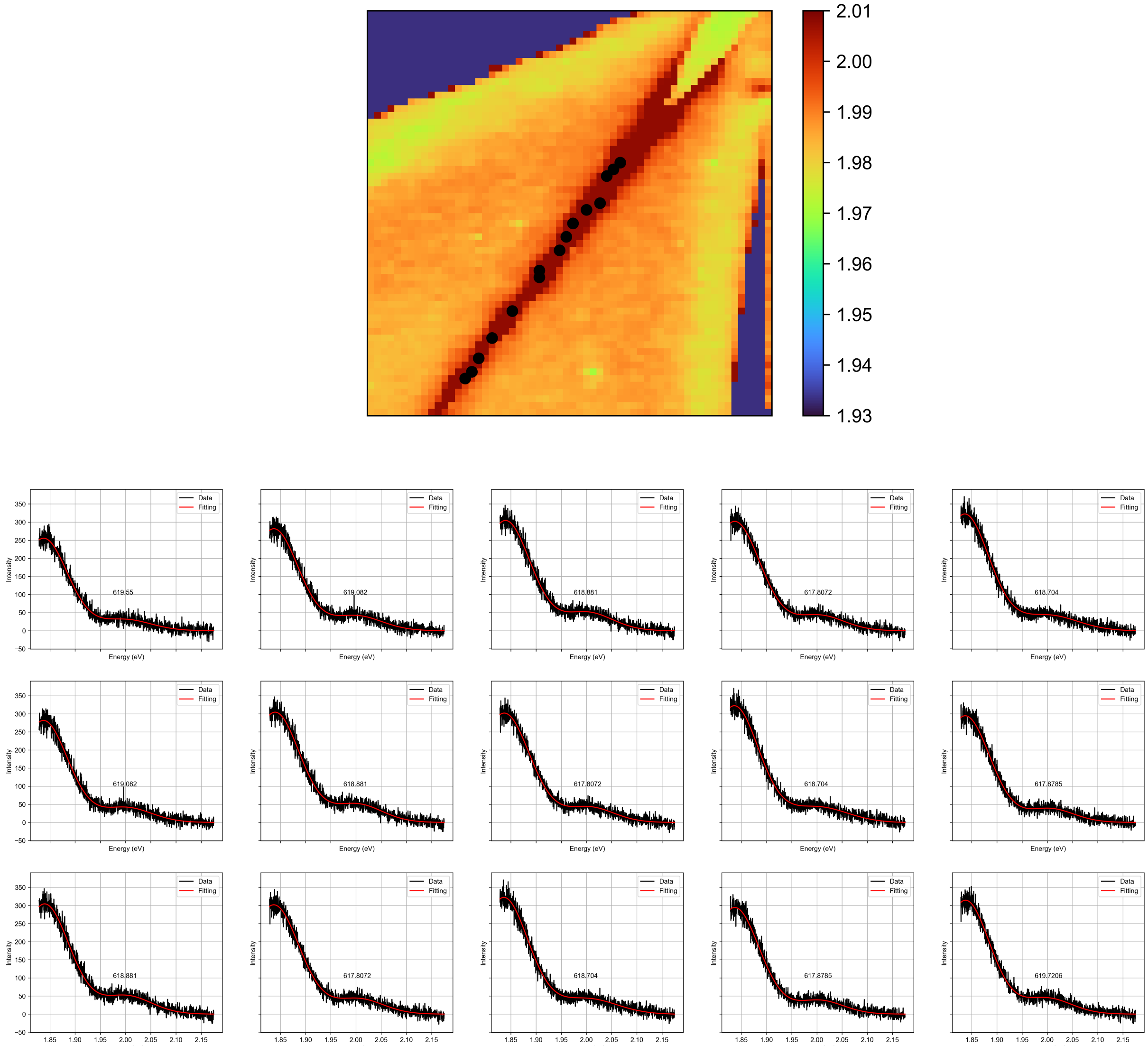

Supplementary Figure S3: **Peak-fitting examples for low-intensity P_2 in doped samples.** The map shown is the same presented in Figure 1, except for the black spots which mark several pixels in which the P_2 is very low. We fitted all spectra with two Gaussian peaks and the fit of position for P_2 is shown for each spectrum. Taking the standard deviation for these values we find uncertainty of ± 3.3 meV, which is well within the experimental spectral resolution of our optical setup.

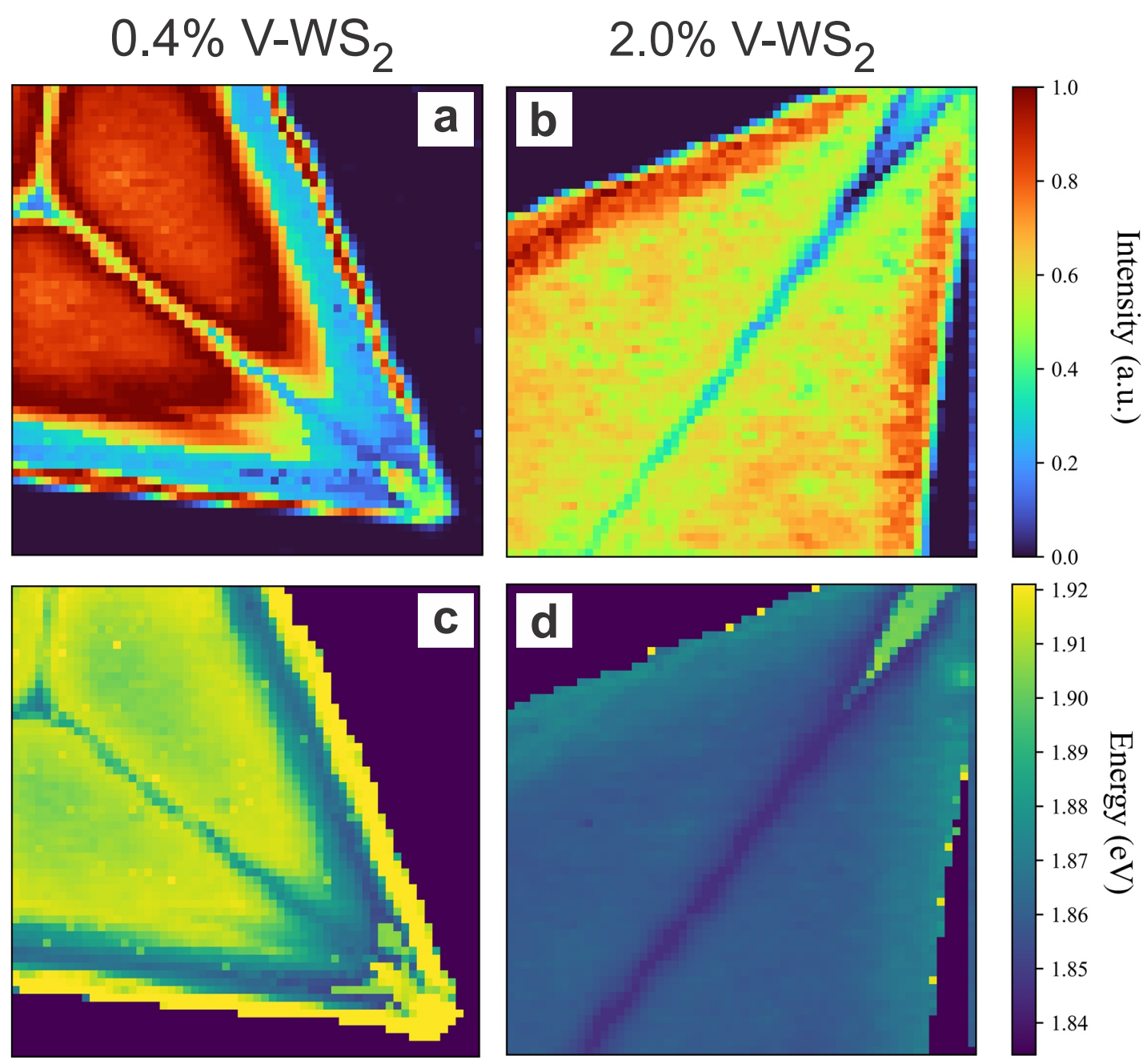

Supplementary Figure S4: **PL mapping of vanadium-doped WS_2 monolayers.** PL intensity maps (integrated area) of P_1 peak for: **a.** 0.4%, and **b.** 2.0% V-doped. PL energy maps of P_1 peak for: **c.** 0.4%, and **d.** 2.0% V-doped. Map area: 20×20 μm^2.

Section 2 Scanning probe microscopy measurements

Scanning probe microscopy (SPM) techniques were employed to further elucidate the structural and electrical differences between pristine and 2.0 at% V-doped WS_2 monolayers. Atomic force microscopy (AFM) in tapping mode reveals that the pristine sample (Figure S5a) maintains a uniform topography, whereas the 2.0 at% doped sample (Figure S5d) displays a prominent elevated line ($\sim$0.6 nm in height) along the defect region.

Electrostatic force microscopy (EFM) of the doped sample (Figure S5e) highlights a dark line at the same location, indicating a higher dielectric constant (*i.e.*, increased metallic character) relative to the surrounding area, an observation absent in the pristine sample (Figure S5b). We infer that vanadium atoms preferentially substitute tungsten atoms along the bisection lines of triangular flakes, leading to localized inhomogeneous doping and the formation of metallic VS_2.

Lateral force microscopy (LFM) further supports this interpretation. The LFM images of the 2.0 at% V-WS_2 sample (Figure S5f) reveal a distinct mechanical contrast along the central line, indicative of compositional differences relative to the rest of the flake. This contrast is not observed in the pristine sample (Figure S5e)[1–3], reinforcing the conclusion that the defect lines possess unique electronic and mechanical properties.

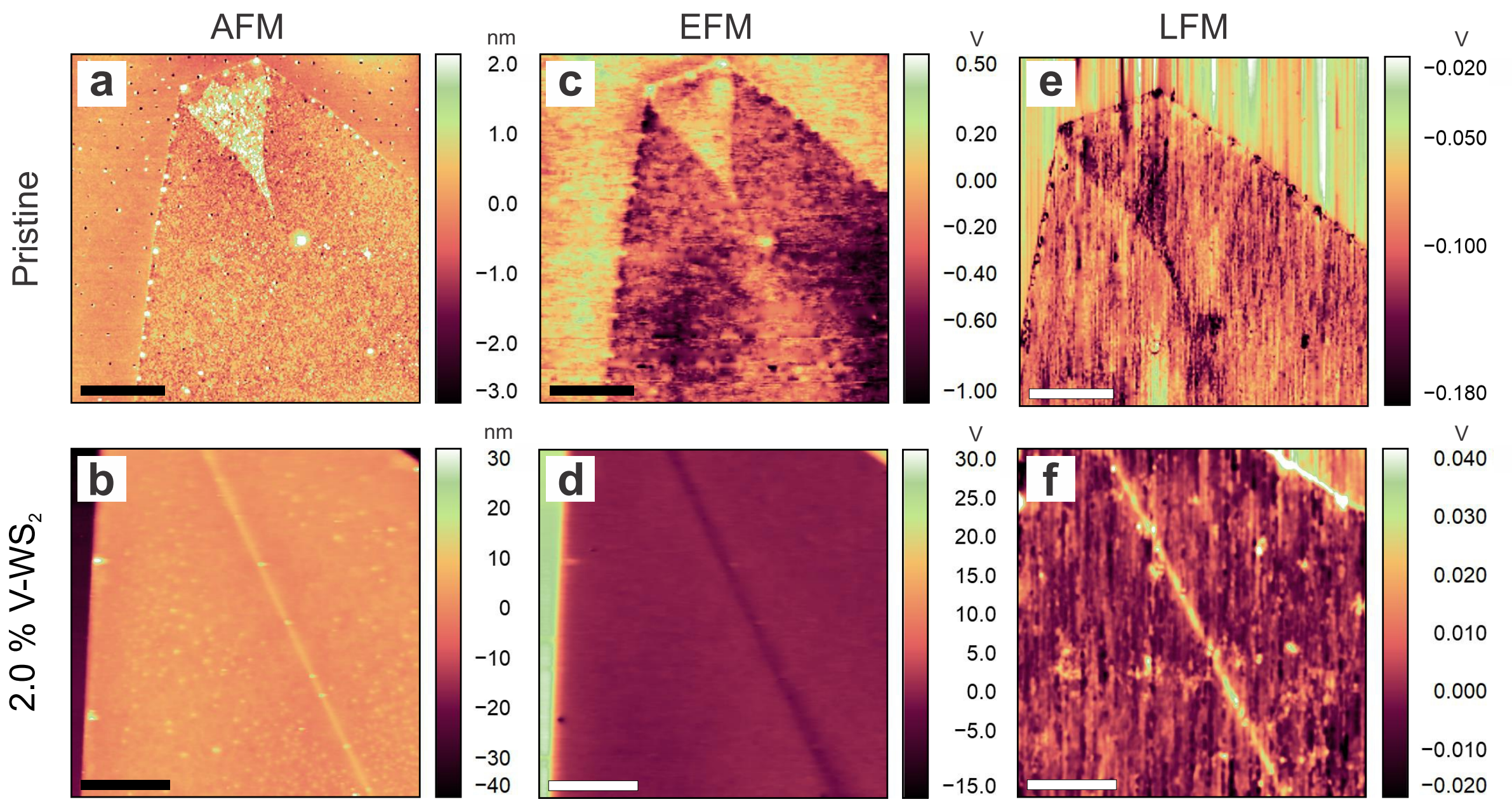

Supplementary Figure S5: **Topographical and Force Microscopy analysis of pristine and vanadium-doped WS_2 monolayers. a,b.** Atomic force microscopy (AFM) images of pristine and 2.0 at% V-doped WS_2, respectively. **c,d.** Electrostatic force microscopy (EFM) images of pristine and 2.0 at% V-doped WS_2, respectively. **e,f.** Lateral force microscopy (LFM) images of pristine and 2.0 at% V-doped WS_2, respectively. Scale bars in all images are 5 µm.

Section 3 Raman frequency and intensity maps

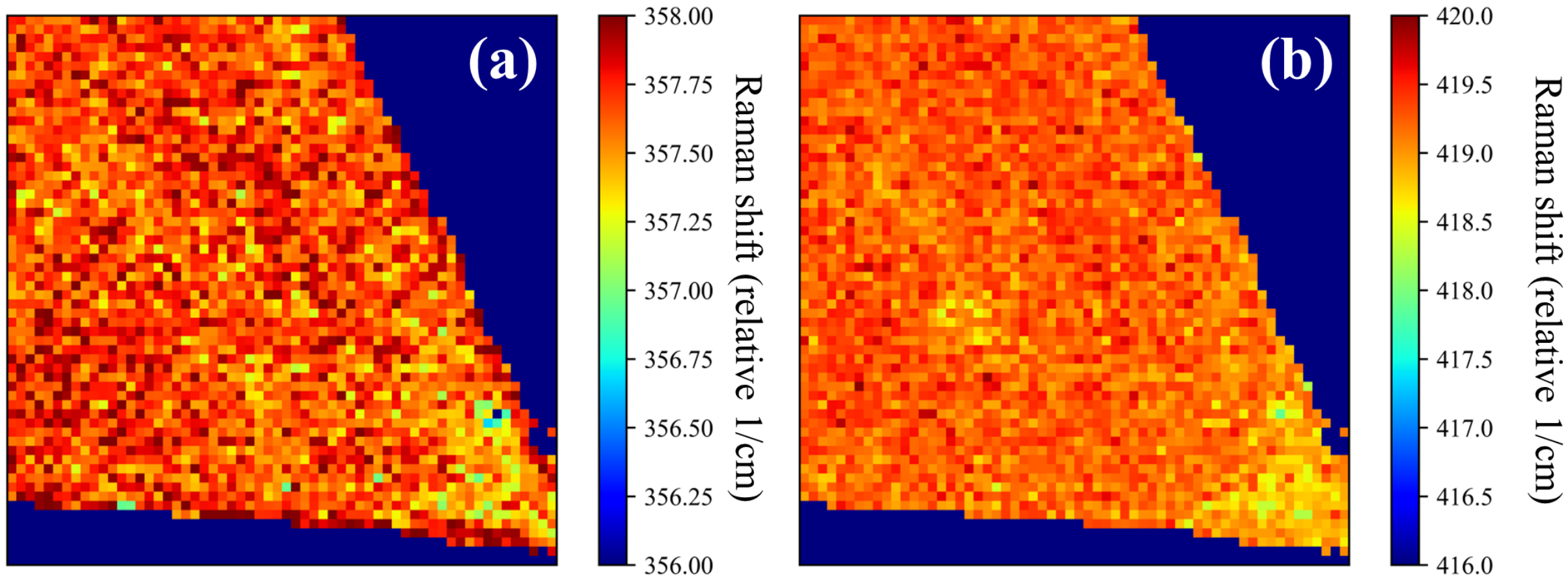

Supplementary Figure S6: **Raman hyperspectral maps of 0.4% vanadium-doped WS_2 monolayer. a.** E' and **b.** A_1' frequency maps for 0.4% V-doped WS_2, respectively. All maps were acquired using a 2.71 eV excitation over a 20×20 μm^2 area.

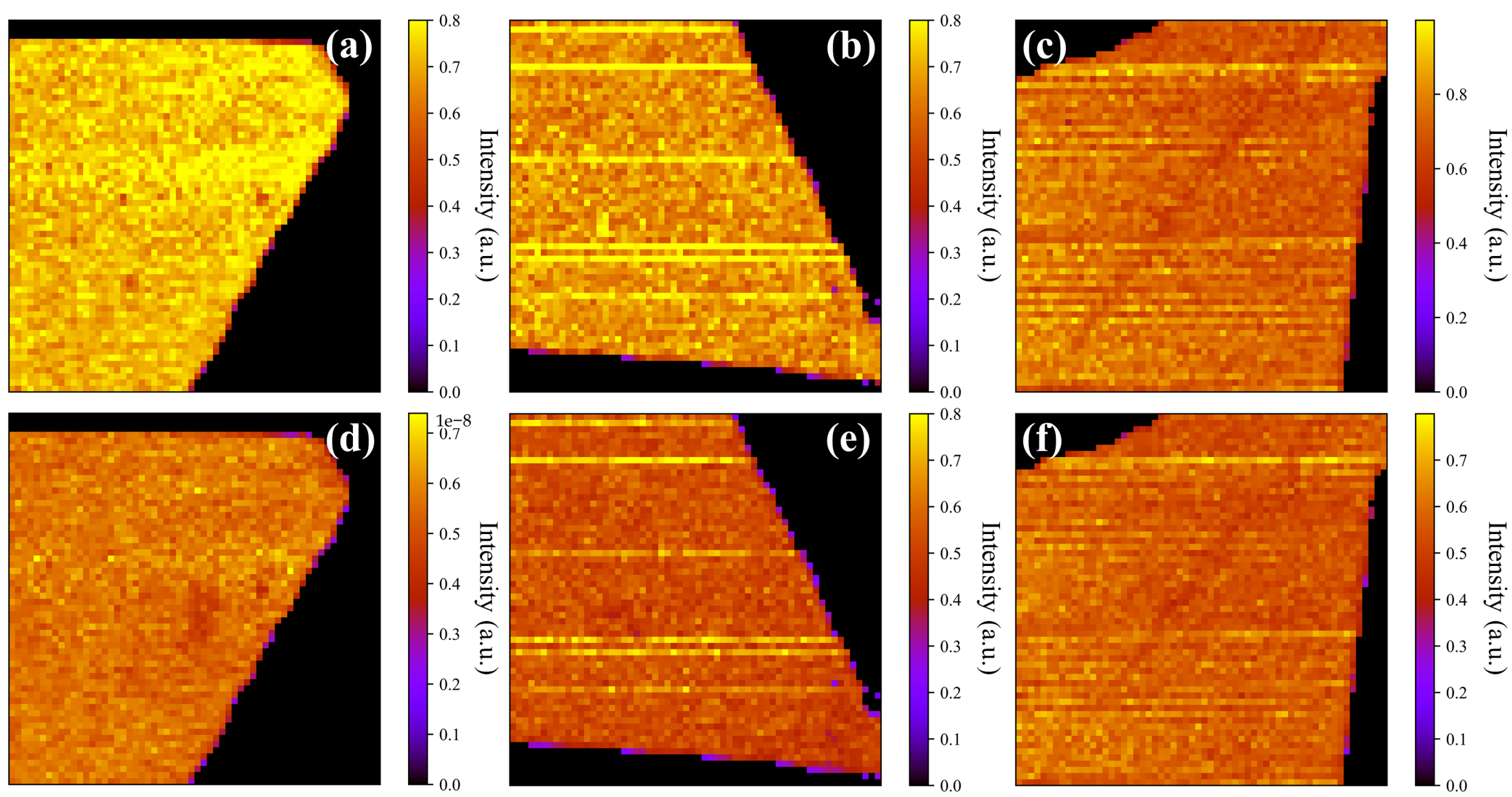

Supplementary Figure S7: **Raman hyperspectral maps of pristine, 0.4%, and 2.0% vanadium-doped WS_2 monolayers.** **(a)** E' and **(d)** A_1' intensity maps for pristine WS_2. **(b)** E' and **(e)** A_1' intensity maps for 0.4% V-WS_2. **(c)** E' and **(f)** A_1' intensity maps for 2.0% at V-WS_2.

Section 4 Resonance-enhanced FWM imaging of pristine WS_2 monolayer

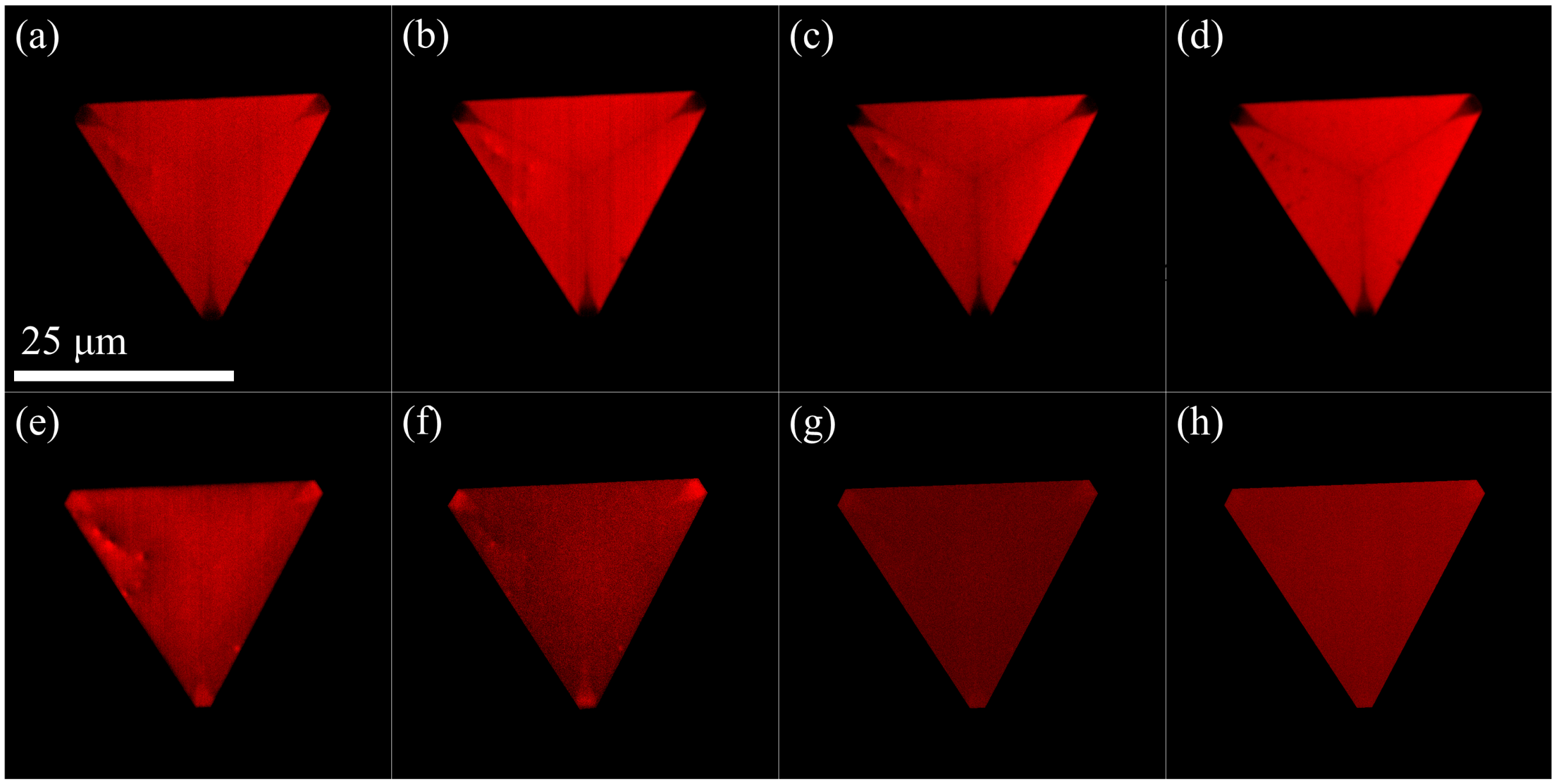

Supplementary Figure S8: **Resonance-enhanced FWM imaging of pristine (0.0% V-doped) WS_2 monolayer.** FWM images at different wavelengths: **(a)** 610, **(b)** 620, **(c)** 626, **(d)** 632, **(e)** 650, **(f)** 667, **(g)** 670, and **(h)** 680 nm. The laser power was kept at ∼70 mW in all measurements (see Methods).

Section 5 Resonance-enhanced FWM imaging of 0.4% V-WS_2 monolayer

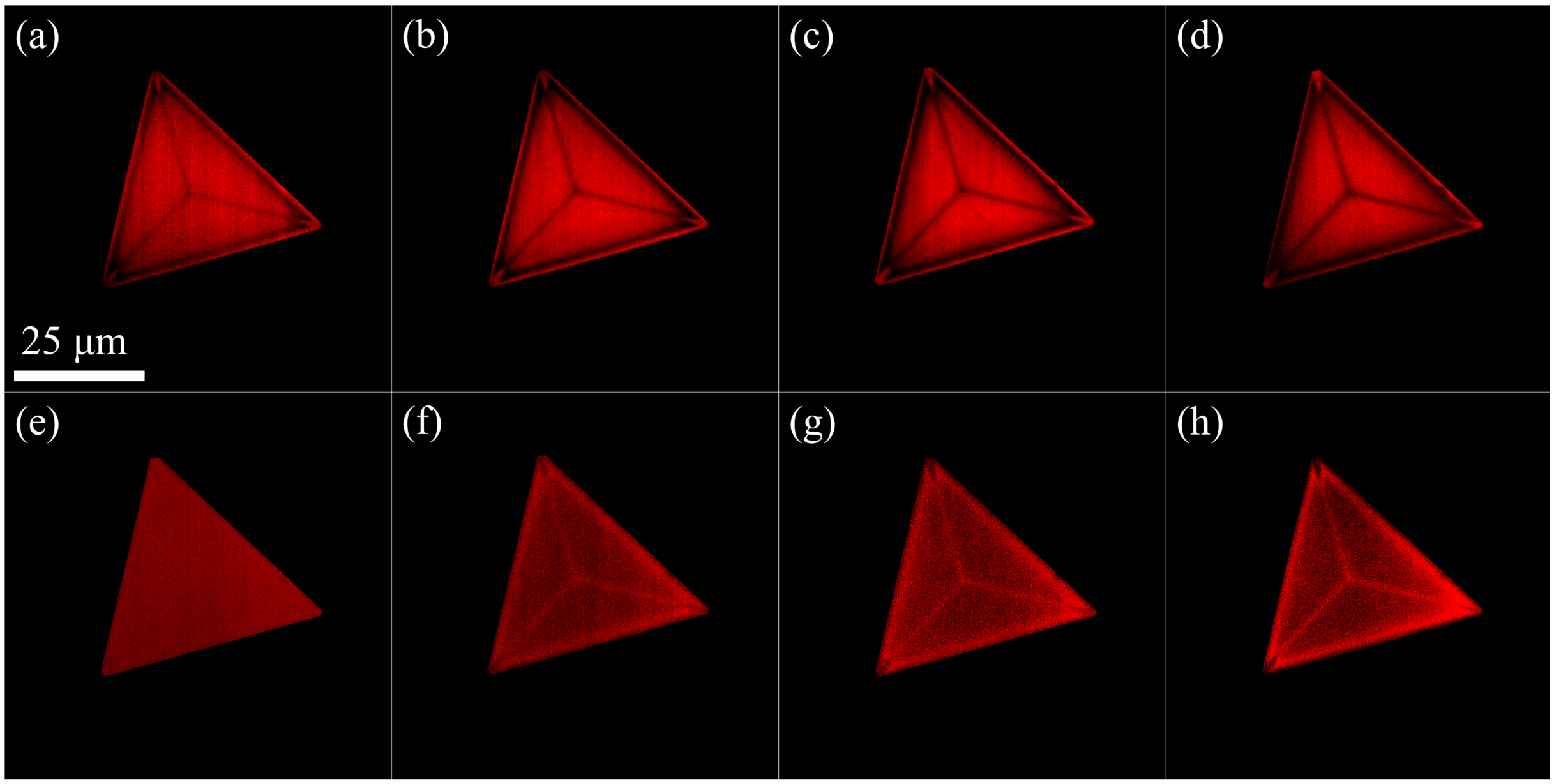

Supplementary Figure S9: **Resonance-enhanced FWM imaging of 0.4% V-doped WS_2 monolayer.** FWM images at different wavelengths: **(a)** 610, **(b)** 620, **(c)** 626, **(d)** 632, **(e)** 650, **(f)** 667, **(g)** 670, and **(h)** 680 nm. The laser power was kept at ∼70 mW in all measurements (see Methods).

Section 6 Resonance-enhanced FWM imaging of 2.0% V-WS_2 monolayer

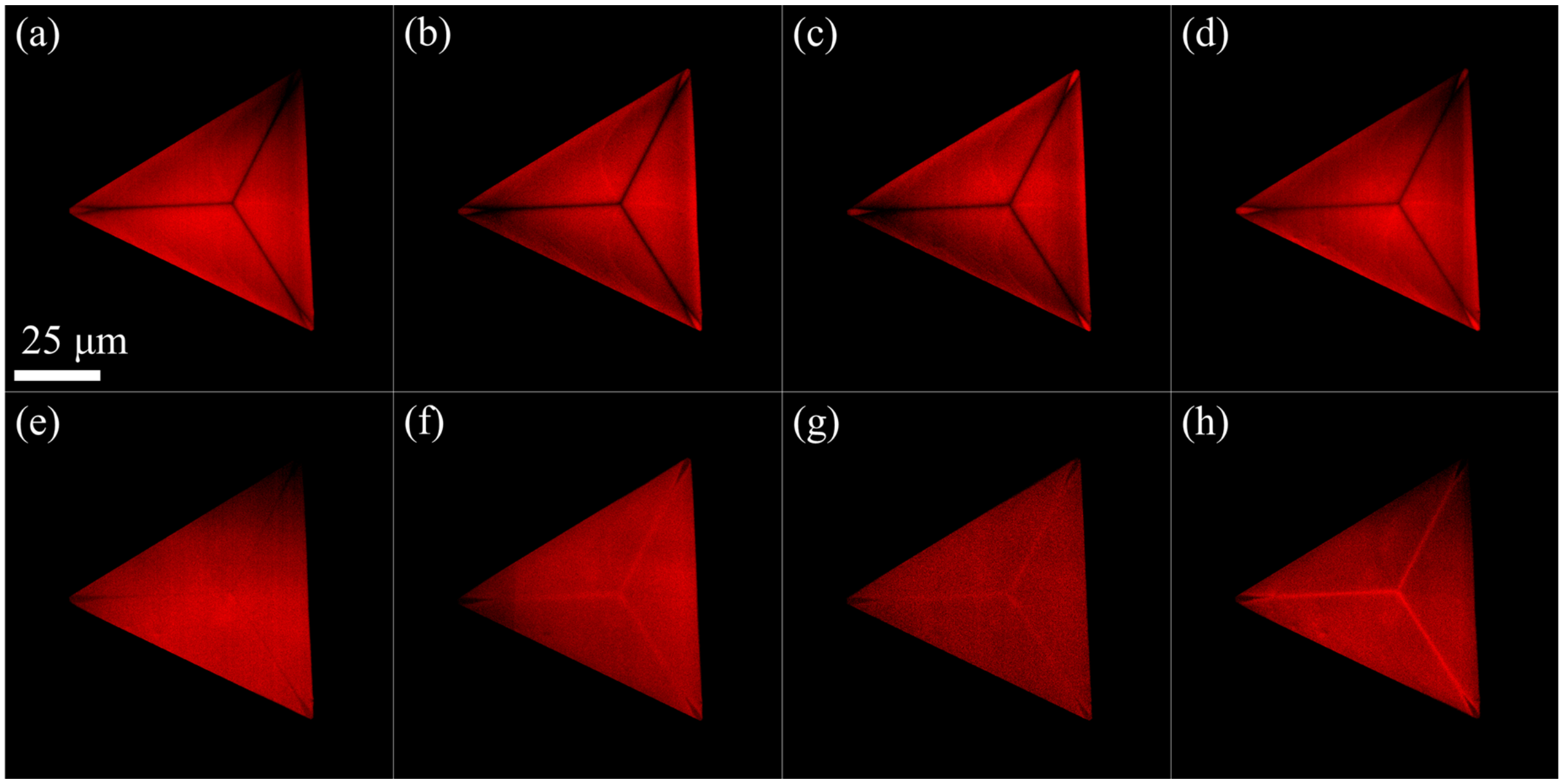

Supplementary Figure S10: **Resonance-enhanced FWM imaging of 2.0% V-doped WS_2 monolayer.** FWM images at different wavelengths: **(a)** 610, **(b)** 620, **(c)** 626, **(d)** 632, **(e)** 650, **(f)** 667, **(g)** 670, and **(h)** 680 nm. The laser power was kept at ∼70 mW in all measurements (see Methods).

Section 7 PL versus NLO contrast

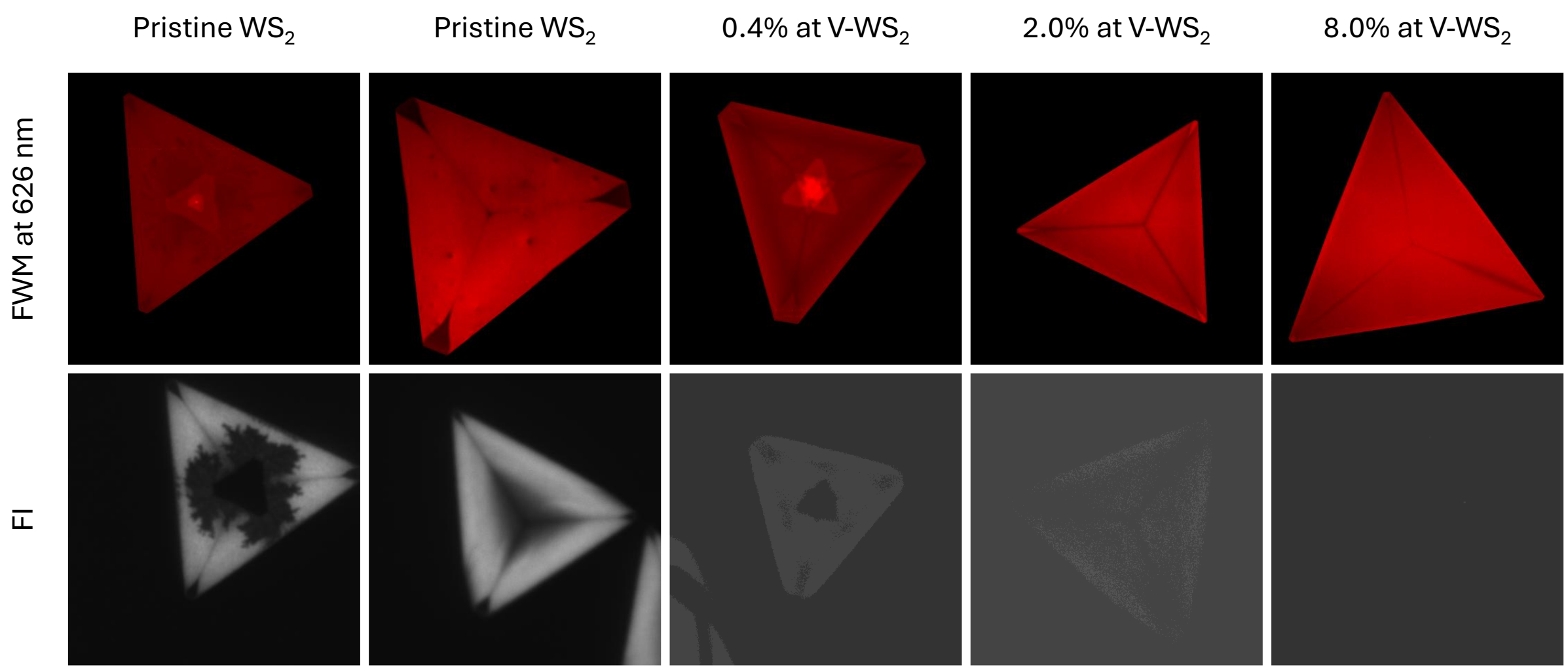

Supplementary Figure S11: **FWM and fluorescence imaging of multiple grown WS_2 flakes.** The upper row depicts the images of different pristine or V-doped WS_2 flakes four-wave-mixing response at 626 nm. The lower row is the FI of the same samples. Doped samples present very low luminescence resulting in very poor signal-to-noise ratio.

Figure SS11 compares a few pristine and V-doped WS_2 monolayers. Even though these measurements were acquired in the same scale of time (approximately 1 minute per image), FWM images present greater contrasts. Additionally, even for slightly doped samples, PL signal decreases significantly. For 2% V-WS_2 flake, for example, signal-to-noise ratio is quite bad to the point we have no information on the flake tips, while in FWM image is very clear that the nonlinear signal is increased for this region in this wavelength.

In order to have high-contrast and a great spatial resolution in PL measurements, hyperspectral PL maps are required. However, these measurements are very time consuming. It is important to note that the FWM images shown in this work cover a large area with approximately 1000 px (lateral size), while PL hyperspectral maps are smaller

and take at least one hour to be acquired.

To quantitatively compare the acquisition efficiency of the two techniques, we note that the high-resolution resonant FWM images (1000 × 1000 pixels, mapping spatial regions from 25 to 100 µm) were recorded in approximately 1 minute using an excitation power of 70 mW (100 µs integration time per pixel). Conversely, linear PL hyperspectral data maps of a limited 60×60 pixel domain (20 × 20 µm^2) required an acquisition window exceeding 1 hour at an excitation power of 150 µW (1 s integration time per pixel). This clearly demonstrates the fast acquisition speed and reduced laser exposure advantages of the FWM technique for mapping defect boundaries.

**Technique**	**FWM**	**Hyperspectral PL**
Surface	(25 µm)^2 up to (100 µm)^2	(20 µm)^2
Acquisition	1000 px by 1000 px	60 px by 60 px
Power	70 mW	150 µW
Total time	30 s up to 60 s ($10^1 - 10^2$ s)	1 hour (10^3 s)
Pixel time	100 µs	1 s

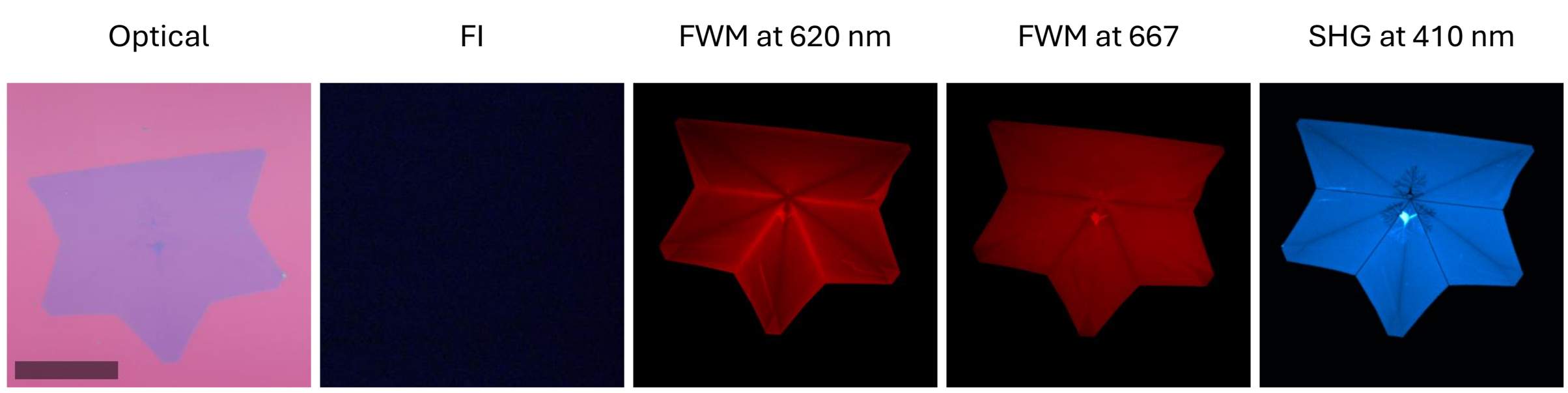

Supplementary Figure S12: **Contrast comparison for multiple techniques applied on a 2% at V-WS$_2$.** Multiple techniques applied in a 2% at V-WS$_2$ that highlights the gain of nonlinear optics techniques in mapping defects, notably defect-resonant FWM in which defect regions change behavior under resonant conditions. Scale bars is 25 µm.

Section 8 Projected band structures

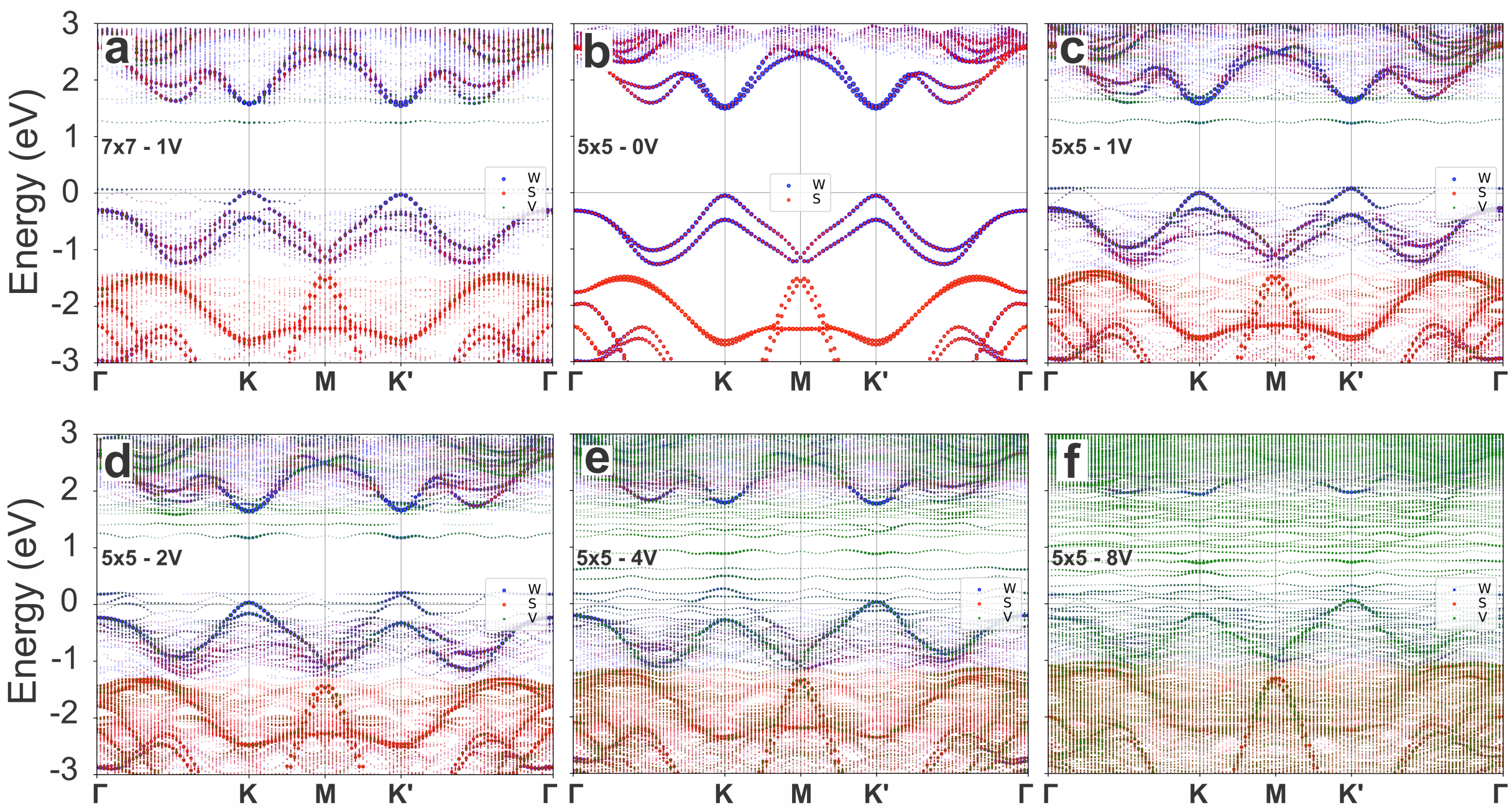

Supplementary Figure S13: **Atom-projected unfolded band structures of pristine and V-doped WS_2 supercells. a.** Unfolded band structure of the 7×7 supercell with one substitutional V atom, corresponding to a V concentration of approximately 2%. **b–f.** Unfolded band structures of the 5×5 supercells with 0, 1, 2, 4, and 8 V atoms, respectively. The size and color of the symbols indicate the atomic projection of each electronic state onto W, S, and V atoms. The V-projected states help identify defect-related bands introduced within or near the pristine band gap, while their evolution with increasing V concentration illustrates the progressive redistribution of spectral weight and the emergence of additional states in the gap region.

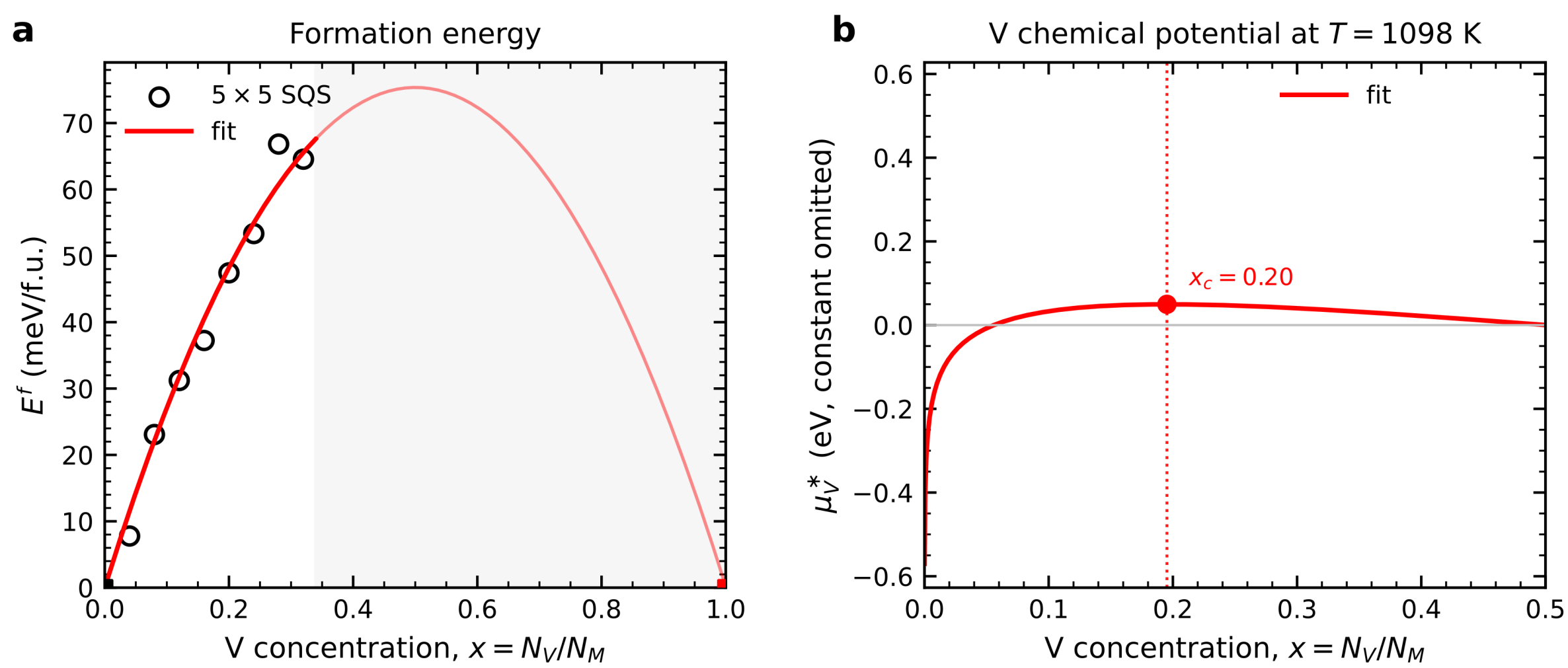

Supplementary Figure S14: **Thermodynamic assessment of V incorporation in WS_2 using the 5 × 5 supercell dataset. a.** Formation energy per formula unit, E_f, as a function of the V concentration, x = NV/NM, calculated from the total energies of substitutionally V-doped WS_2 supercells relative to the WS_2 and VS_2 end members. The data were fitted using the quadratic form $E_f(x) = ax+bx^2$. The shaded region indicates extrapolation beyond the explicitly calculated concentration range. **b.** Corresponding relative V chemical potential, $\mu_V^*(x,T) = a+2bx+k_BT\ln[x/(1-x)]$, evaluated at the growth temperature T = 1098 K. The constant offset $E_{VS_2} - E_{WS_2}$ is omitted because it only shifts the chemical potential vertically and does not affect the critical concentration.

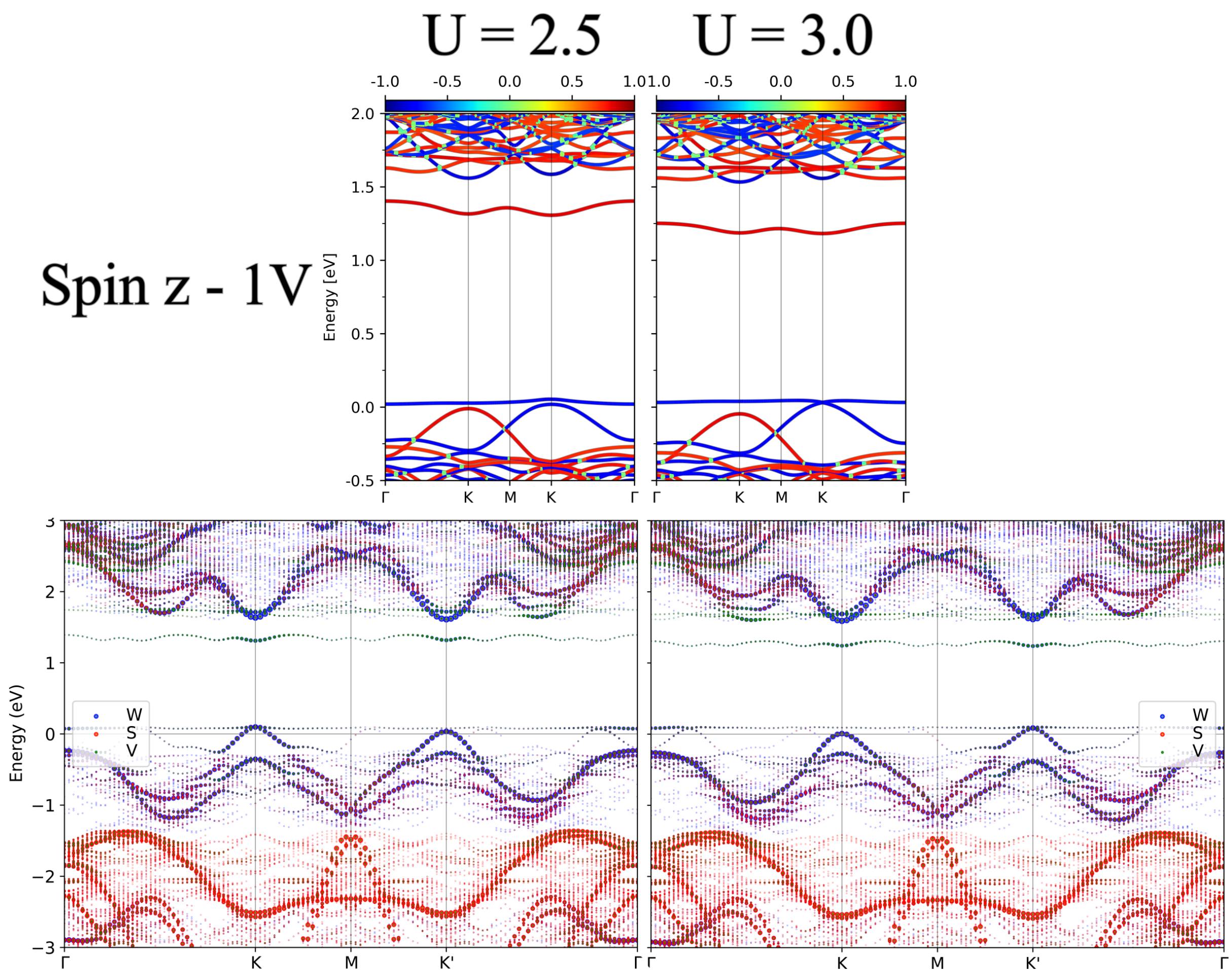

Supplementary Figure S15: **Comparison of the electronic structure of V-doped WS_2 calculated with $U = 2.5$ eV and $U = 3.0$ eV.** The calculations were performed for a 5 × 5 WS_2 supercell containing one substitutional V atom. Upper panels show the spin-resolved folded band structures, with the color scale indicating the out-of-plane spin projection, S_z. Lower panels show the atom-projected unfolded band structures, where the size and color of the symbols indicate the projection onto W, S, and V atoms. The energy scale is referenced to the Fermi level, $E_{\mathrm{Fermi}} = 0$ eV. The two U values lead to only small quantitative changes, while preserving the qualitative V-induced defect/band-edge hybridized states.

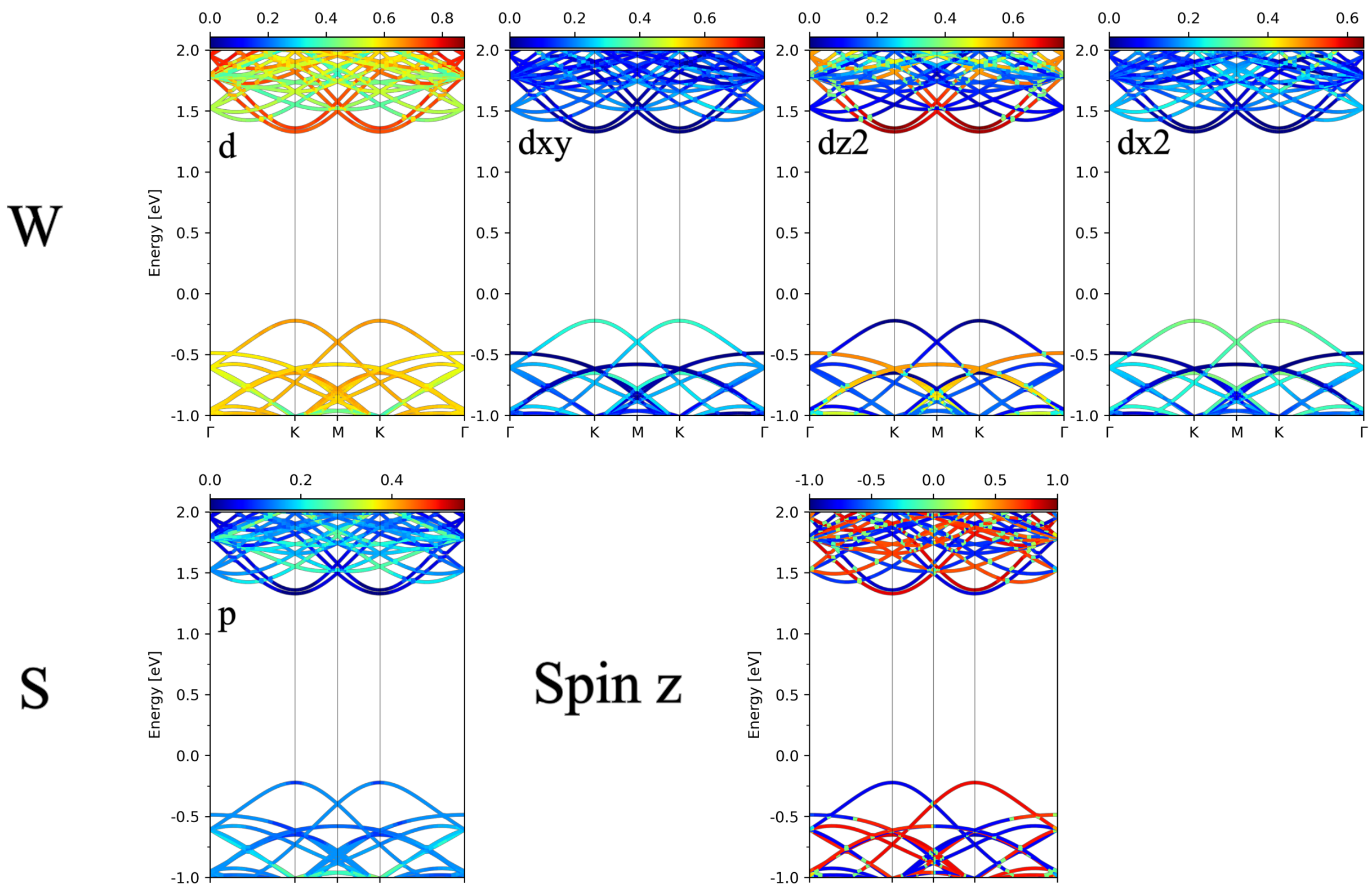

Supplementary Figure S16: **Projected band structure of a 5×5 WS_2 supercell including spin–orbit coupling (SOC).** Orbital-resolved projections are shown for tungsten (d, d_{xy}, d_{z^2}, and $d_{x^2-y^2}$) and sulfur (p) states. Additionally, the projection onto the z component of the electron spin is presented.

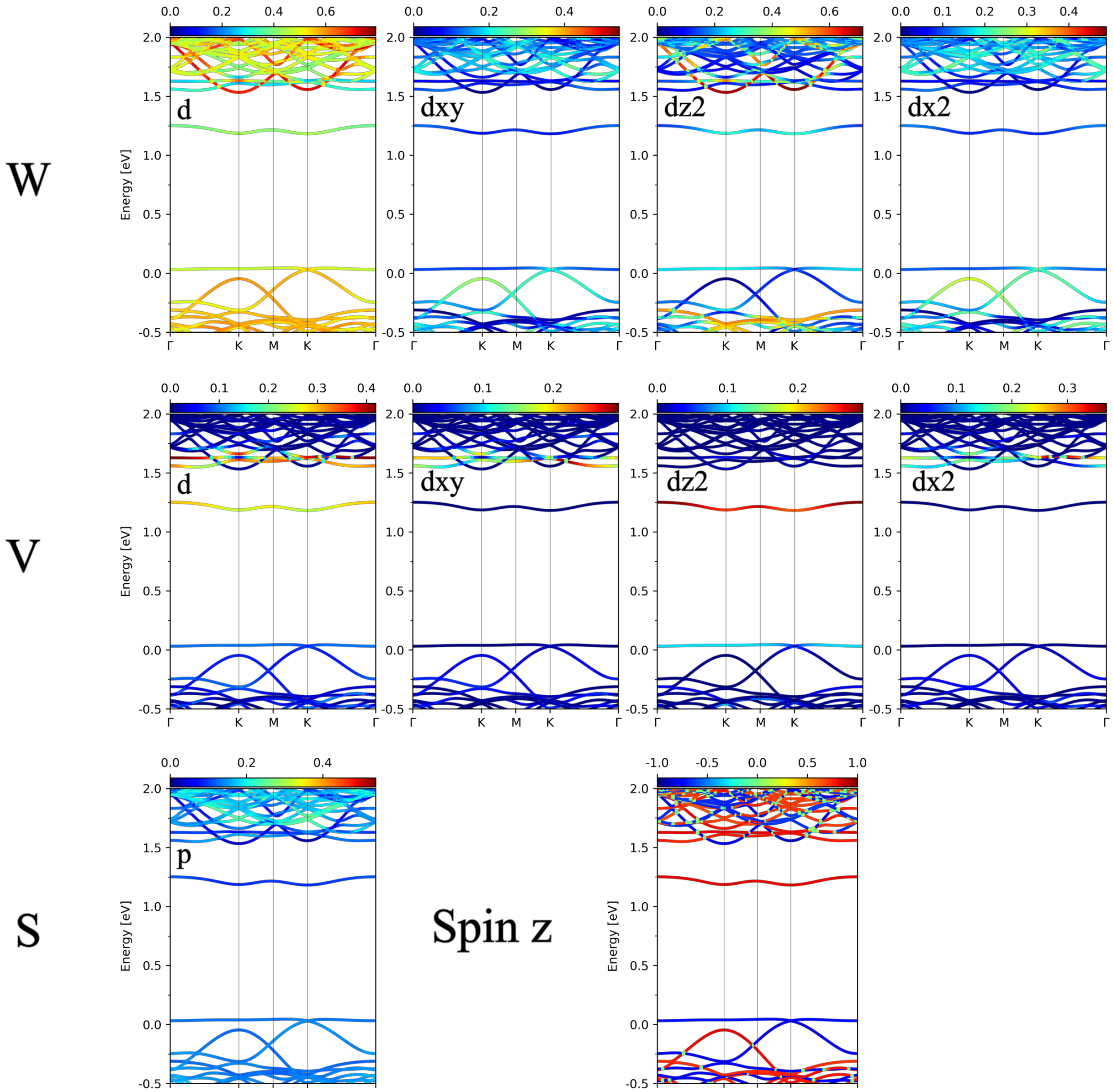

Supplementary Figure S17: **Projected band structure of a 5×5 WS_2 supercell with a single V substitution ($x = 0.04$).** Orbital-resolved projections are shown for tungsten (d), sulfur (p), and vanadium (d) states. The specific contributions from d_{xy}, d_{z^2}, and $d_{x^2-y^2}$ orbitals are also included. The projection onto the z component of the electron spin is also presented.

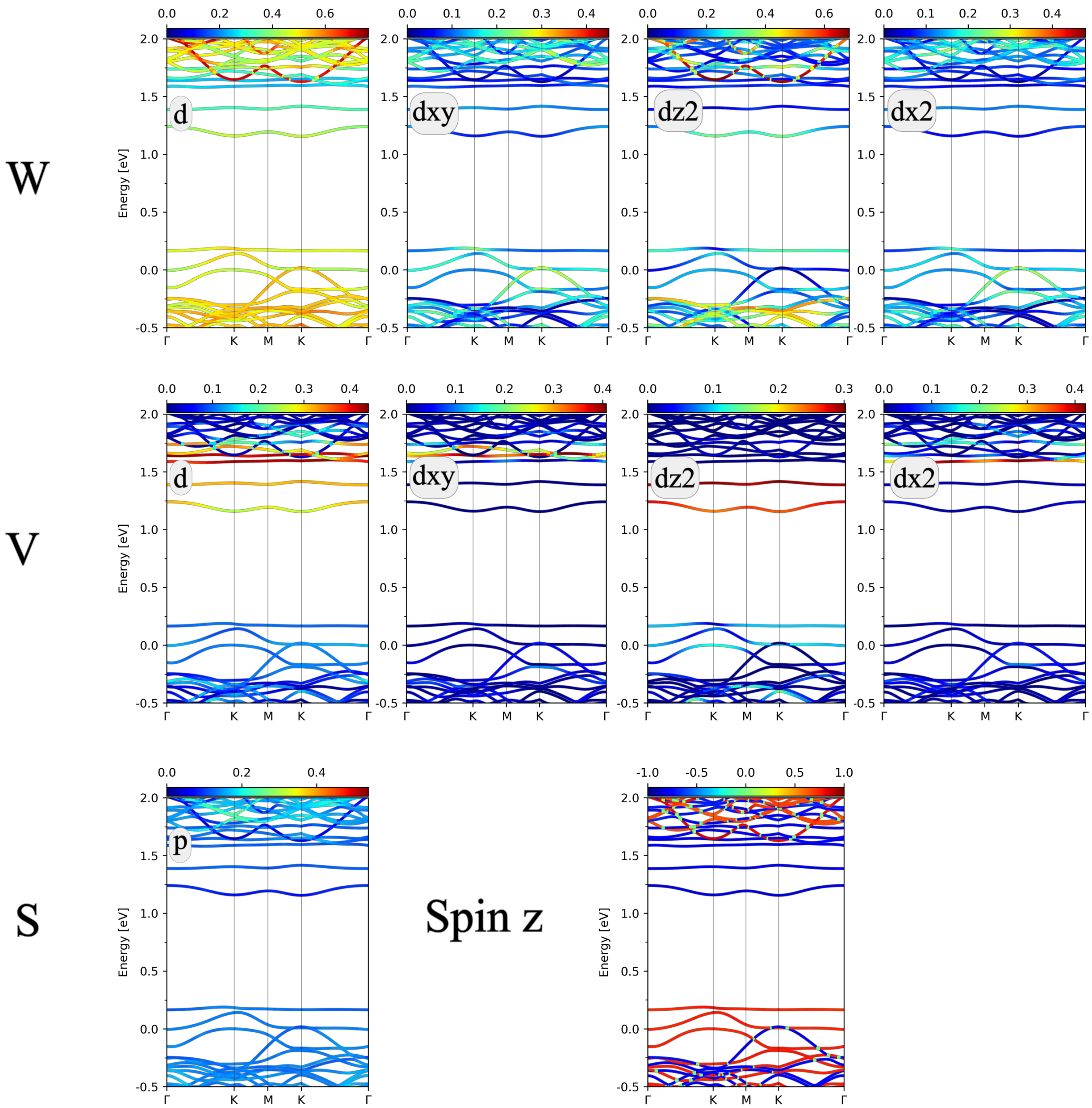

Supplementary Figure S18: **Projected band structure of a 5×5 WS_2 supercell with two V substitutions ($x = 0.08$).** Orbital-resolved projections are shown for tungsten (d), sulfur (p), and vanadium (d) states. The specific contributions from d_{xy}, d_{z^2}, and $d_{x^2-y^2}$ orbitals are also included. The projection onto the z component of the electron spin is also presented.

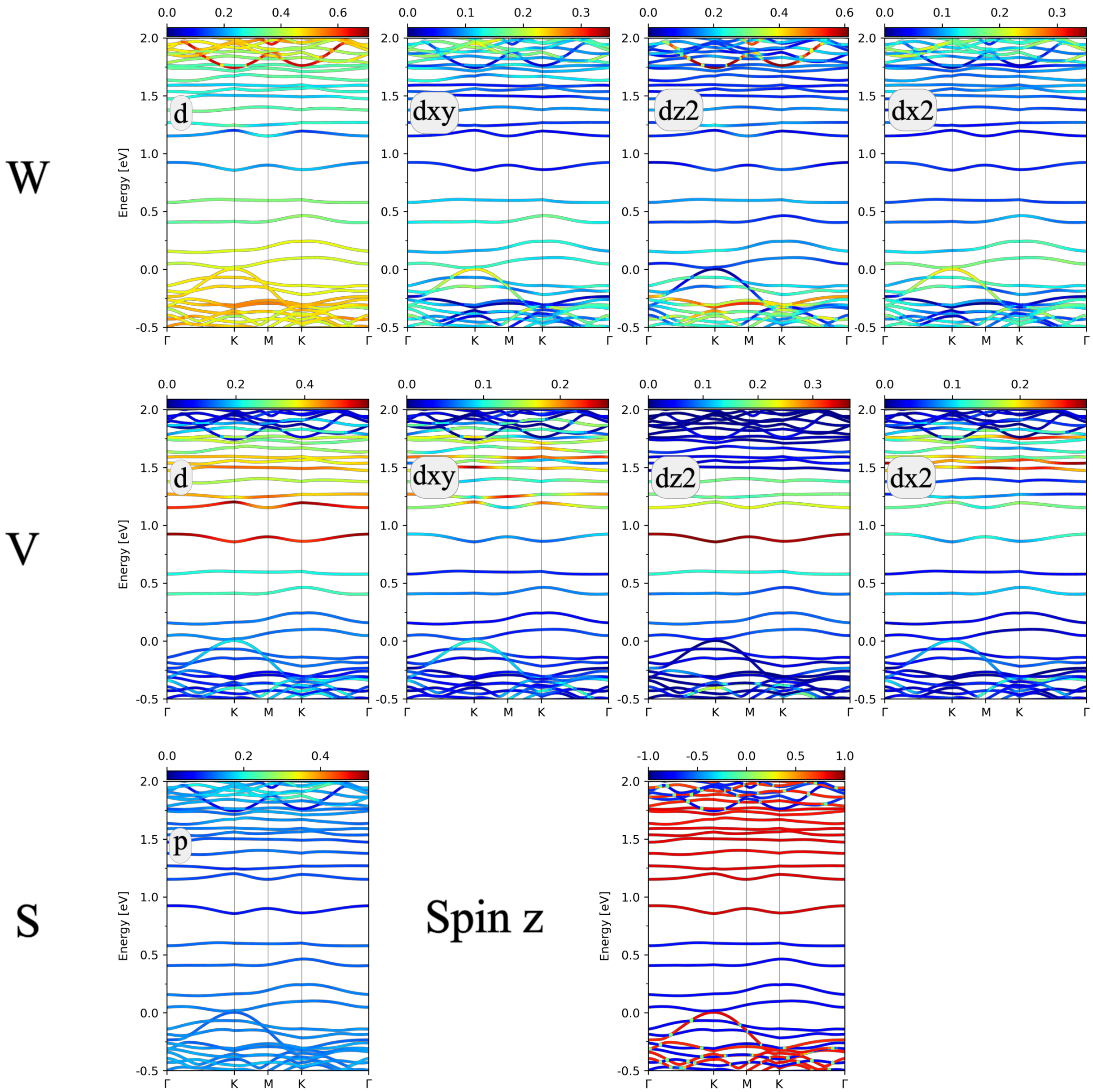

Supplementary Figure S19: **Projected band structure of a 5×5 WS_2 supercell with four V substitutions ($x = 0.16$).** Orbital-resolved projections are shown for tungsten (d), sulfur (p), and vanadium (d) states. The specific contributions from d_{xy}, d_{z^2}, and $d_{x^2-y^2}$ orbitals are also included. The projection onto the z component of the electron spin is also presented.

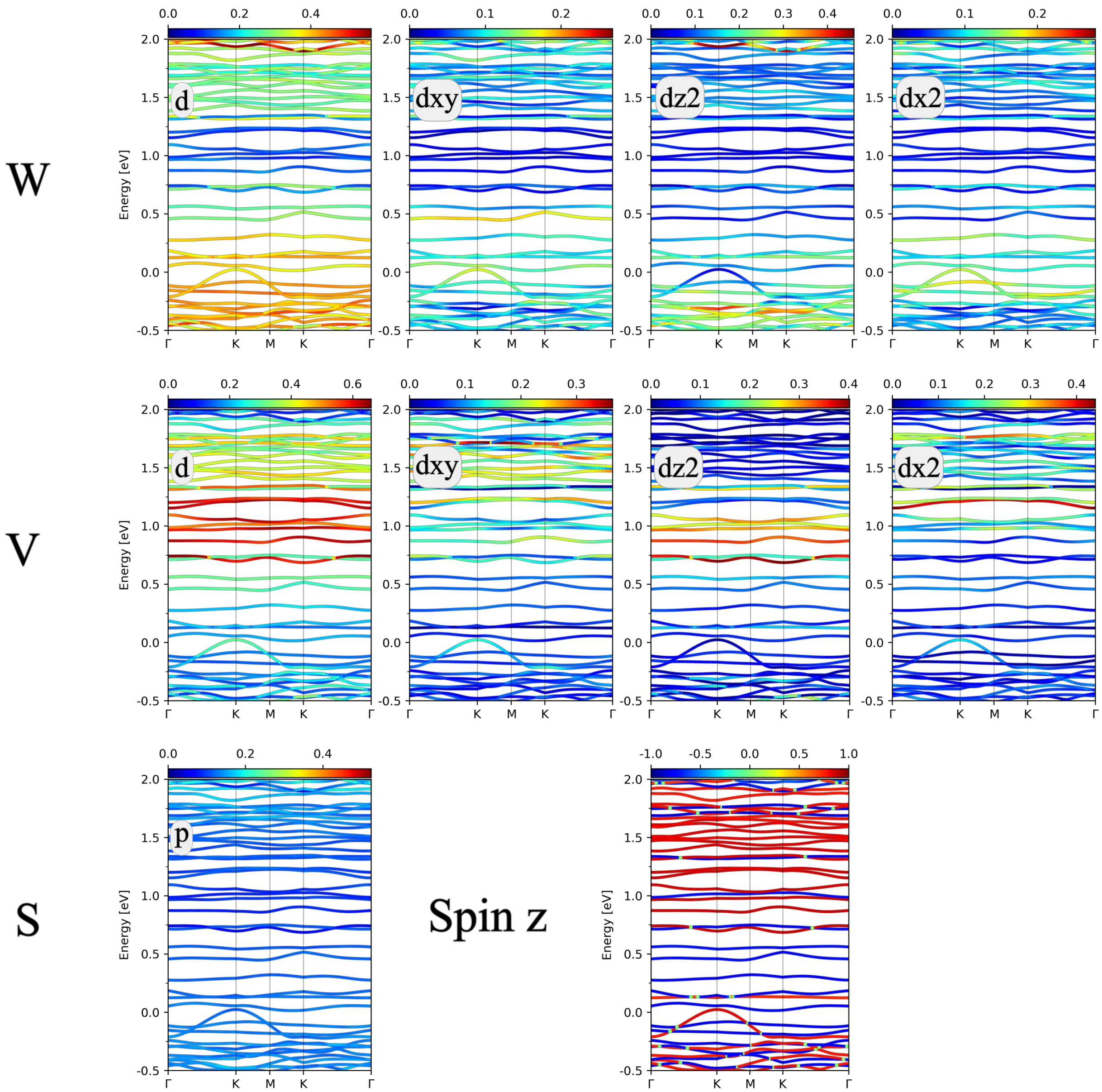

Supplementary Figure S20: **Projected band structure of a 5×5 WS_2 supercell with eight V substitutions ($x = 0.32$).** Orbital-resolved projections are shown for tungsten (d), sulfur (p), and vanadium (d) states. The specific contributions from d_{xy}, d_{z^2}, and $d_{x^2-y^2}$ orbitals are also included. The projection onto the z component of the electron spin is also presented.

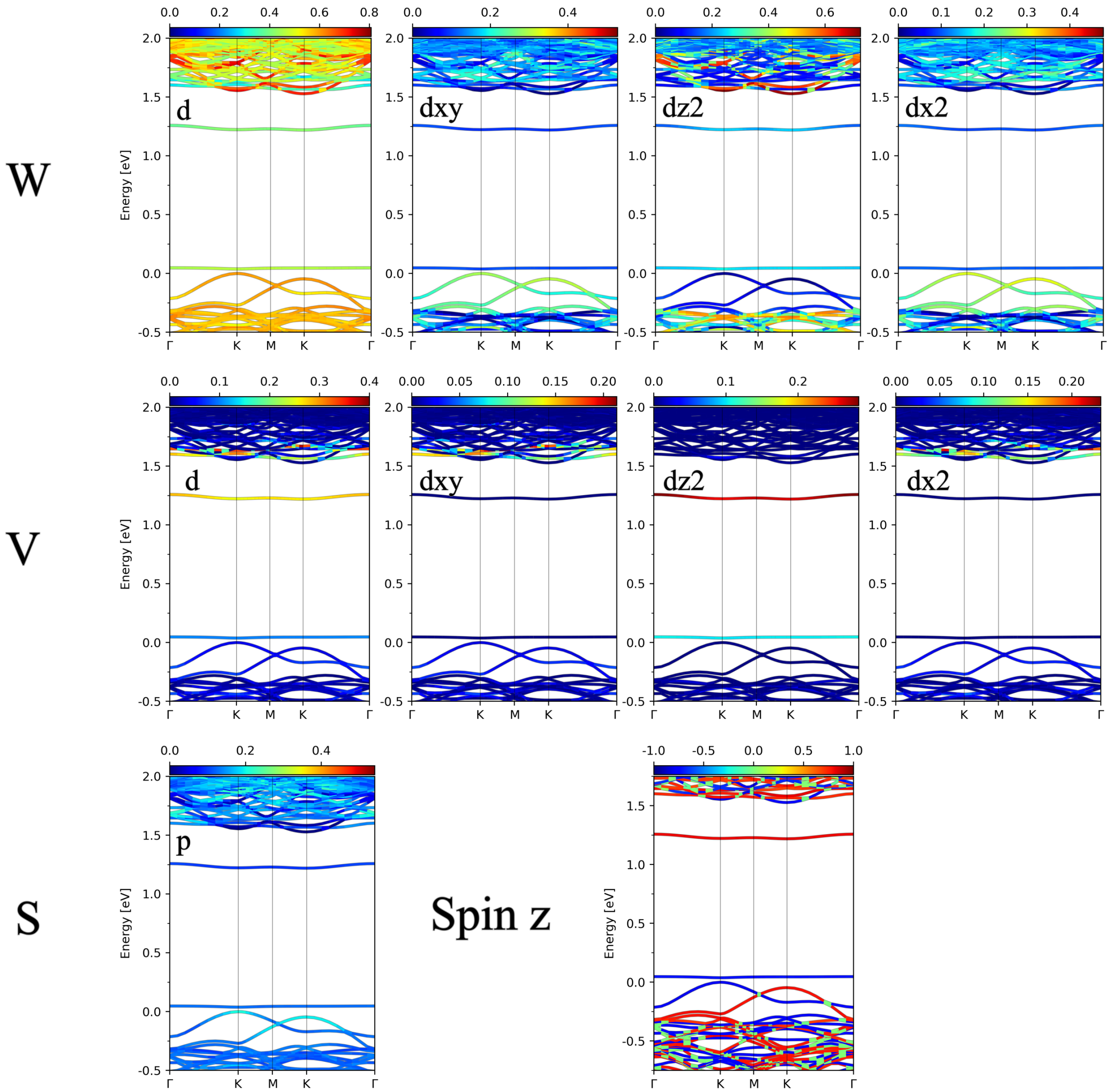

Supplementary Figure S21: **Projected band structure of a 7×7 WS_2 supercell with one V substitution ($x = 0.02$).** Orbital-resolved projections are shown for tungsten (d), sulfur (p), and vanadium (d) states. The specific contributions from d_{xy}, d_{z^2}, and $d_{x^2-y^2}$ orbitals are also included. The projection onto the z component of the electron spin is also presented.

Section 9 Fluorescence imaging

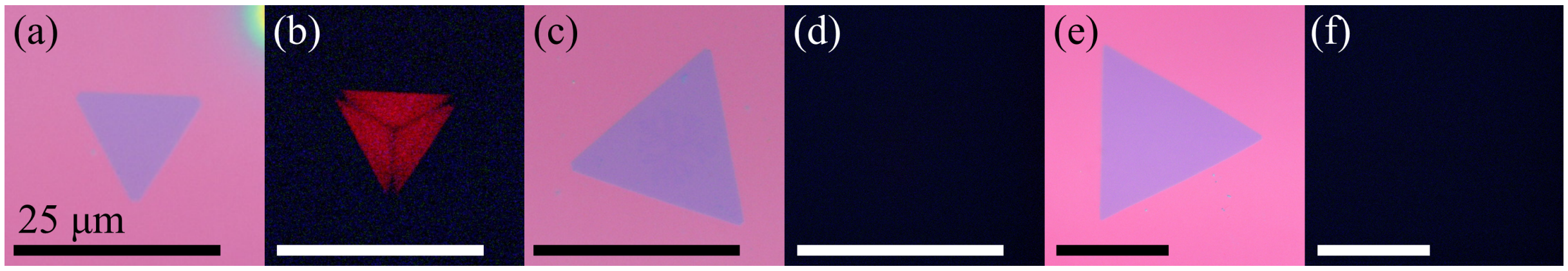

Supplementary Figure S22: **Fluorescence imaging.** Optical and fluorescent images of **(a,b)** pristine WS_2, **(c,d)** 0.4%, and **(e,f)** 2.0% at V-WS_2 flakes. Scale bars are 25 μm.

Section 10 Tunable FWM laser excitation

Table S1: **FWM Operating Parameters and Filter Wavelengths.** Details of the tunable pump wavelengths (ω_1) required to generate the specific FWM signal wavelengths ($\omega_{\mathrm{FWM}} = 2\omega_1 - \omega_2$) using the fixed 1064 nm laser (ω_2) that resonate with the WS_2 excitonic states, alongside the specific optical filters employed for each excitation beam.

Pump (nm)	775	783	788	793	807	820	822	830
FWM (nm)	610	620	626	632	650	667	670	680
Filter (nm)	605/10	620/20	632/22	632/22	655/40	661/11	670/10	680/13
FWM (eV)	2.03	1.99	1.98	1.96	1.90	1.86	1.85	1.82

Section 11 FWM delay dependency

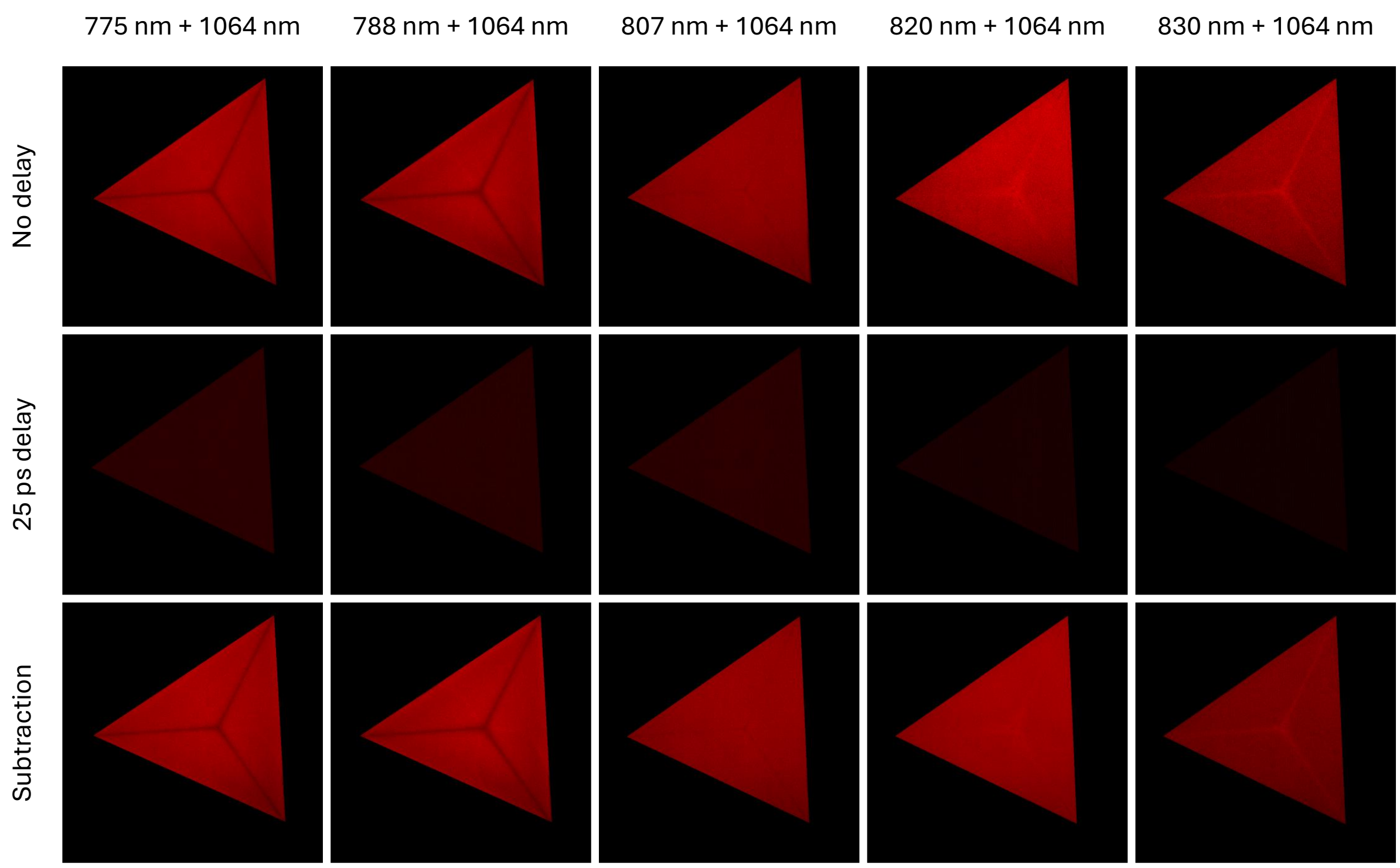

Supplementary Figure S23: **FWM delay dependency.** FWM imaging of the flake shown in Figure SS10 at different excitation wavelengths. The first row depicts the images acquired using both beams (as described in Methods) with no delay. The second row is the result of applying a 25 ps delay between the beams. The third row is the difference between both images.

To ensure that the four-wave-mixing measurements didn't display any contribution from other optical phenomena – such as two-photon PL, for example – we used different filters for each wavelength range as reported in Table S1. Additionally, for every FWM measurement, we performed the same experiment with identical parameters except for a 25 ps delay between the pump and the 1064 nm beam. The second step measures all optical phenomena within the filter range that is not FWM. Lastly, we subtract both images to obtain a pure FWM image. This protocol was applied to every FWM image in

this work.

Section 12 Second harmonic generation imaging

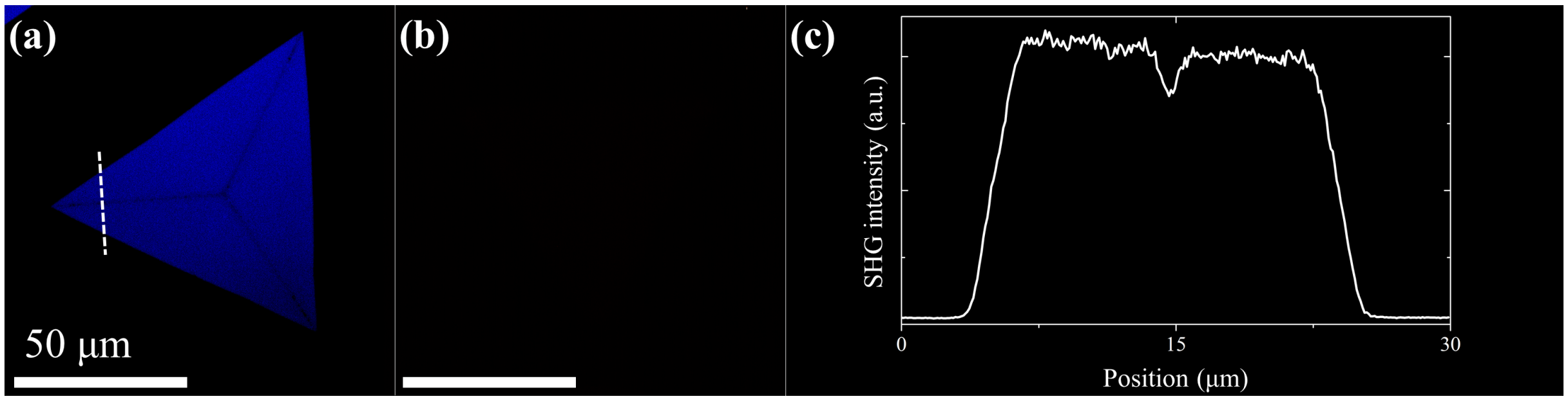

Supplementary Figure S24: **Second harmonic generation (SHG) imaging.** SHG images of 2.0% V-WS_2 with pump laser at **(a)** 810 nm (SHG at 405 nm) and **(b)** 790 nm (SHG at 395 nm). Both images were acquired using the same 405/10 nm filter. This panel yields a true zero-intensity background because the resulting second harmonic emission at 395 nm is entirely blocked by the 405/10 nm bandpass filter, thus confirming that the mesured signal at 405 nm is SHG and not two-photon PL for example. **(c)** Line profile of the SHG signal for 405 nm at the dashed line shown in (a).

Section 13 Additional resonant FWM examples

To ensure that the four-wave-mixing resonant behavior with the defective state was observed consistently over the rest of the sample, we measured different flakes with different concentrations. We applied the same protocol (see Methods) to the flakes discussed in the main text to observe the same behavior.

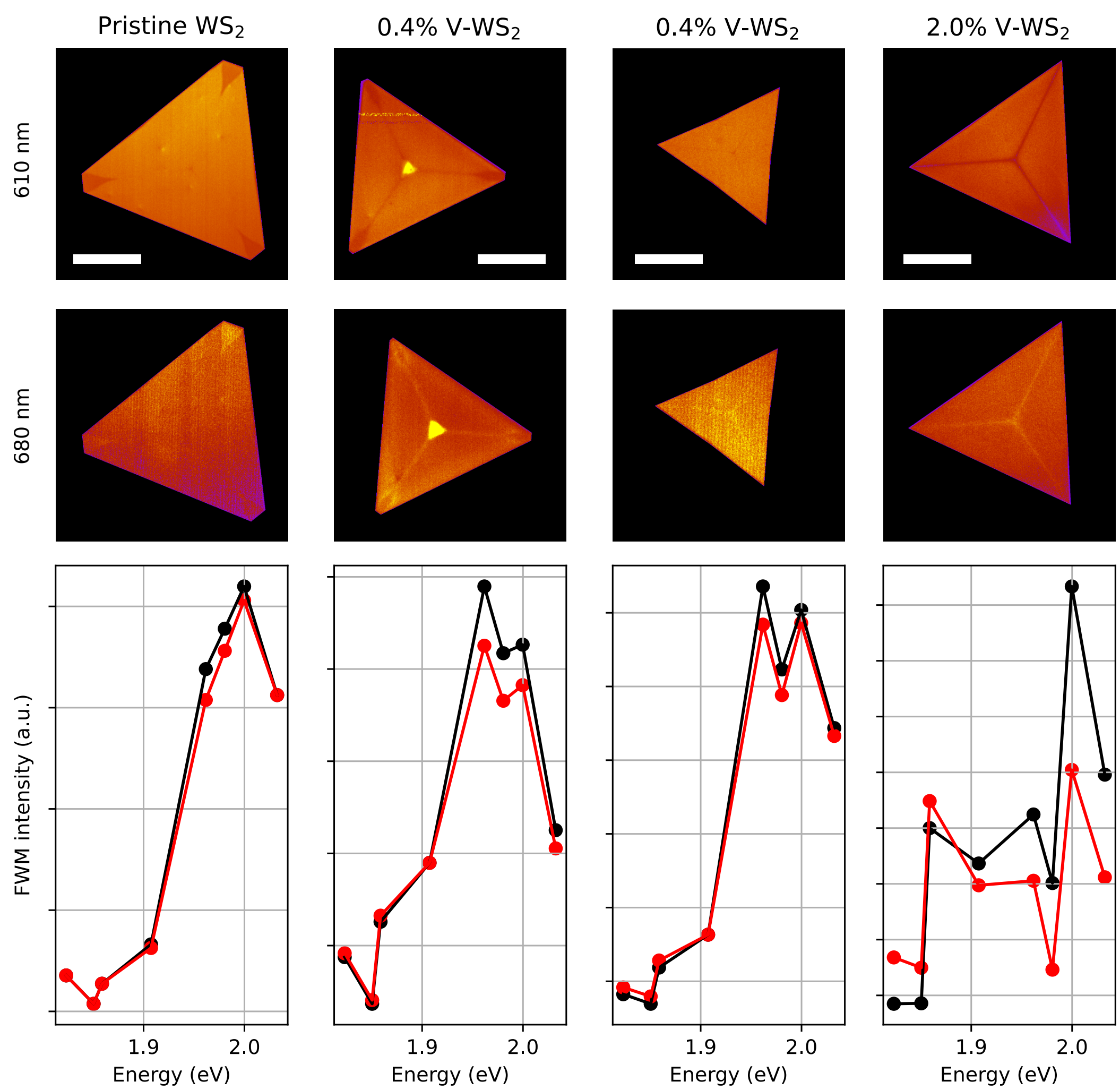

Supplementary Figure S25: **Additional FWM resonant profiles.** FWM P_1 (680 nm) and P_2 (610 nm) resonances for different flakes at different V concentrations. Their corresponding FWM resonant profiles are shown below at the DL (red) and at monolayer region (black).

Section 14 FWM spatial resolution

The spatial resolution was determined using the knife-edge method by extracting an intensity line profile across a sharp edge from the FWM image (Figure 3b). Taking the first derivative of this profile yields the line spread function of our optical setup. Fitting this derivative with a Gaussian profile yields a width parameter of $w = 0.224 \pm 0.016$ µm. This translates to a Full Width at Half Maximum (FWHM $= 2\sqrt{2 \ln 2} w$) of 0.528 ± 0.037 µm, defining the spatial resolution of our setup.

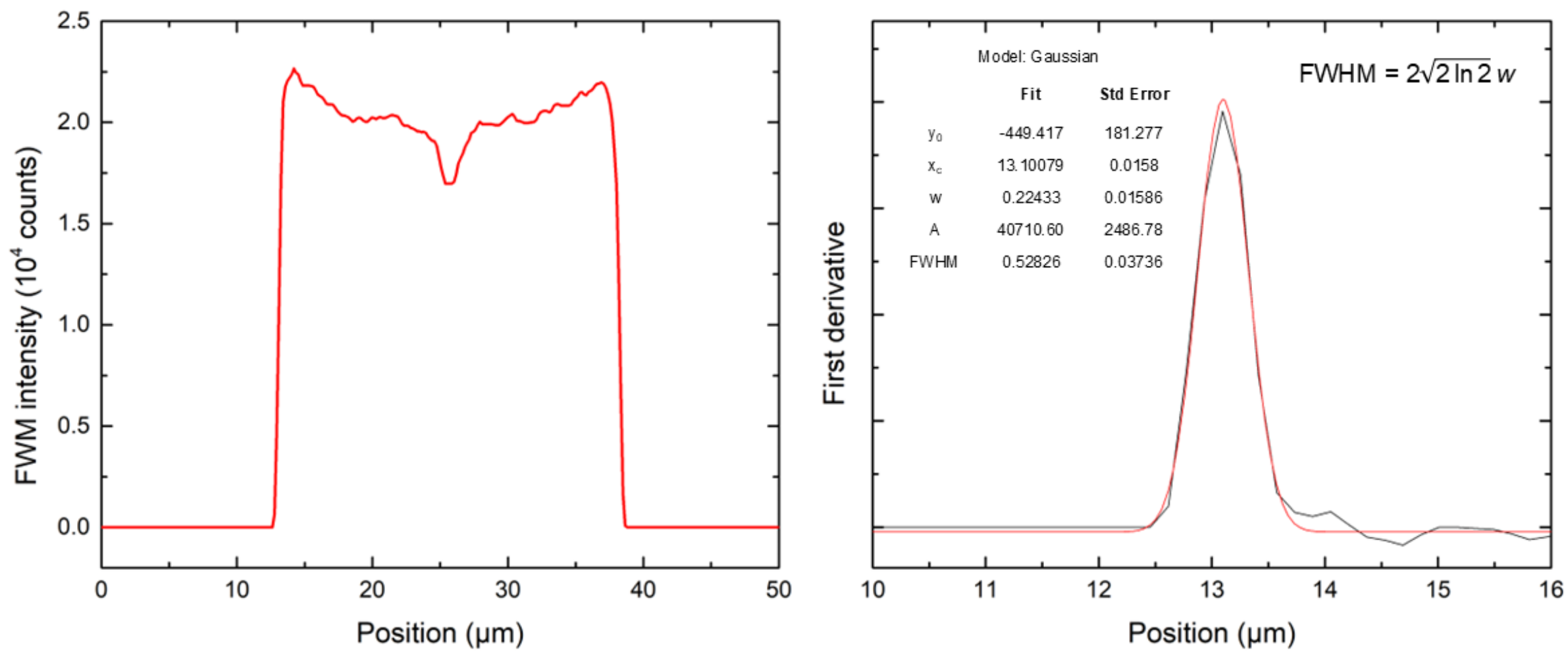

Supplementary Figure S26: **Knife-edge method applied to FWM intensity profile.** (left) FWM intensity profile for 2.0% at V-WS_2 at 610 nm (Figure 3). (right) The first derivative of the FWM profile (left portion) and the Gaussian fit.

References

[1] Weiss, P. S. Scanning probe microscopy and spectroscopy. theory, techniques, and applications. 2nd edition edited by d. w. bonnell (university of pennsylvania). wiley-vch: New york. 2001. xiv + 494 pp. $130. isbn 0-471-24824-x. (2001).

[2] Yu, Y.-J. *et al.* Tuning the Graphene Work Function by Electric Field Effect. *Nano Letters* **9**, 3430–3434 (2009).

[3] Araujo, K. A. *et al.* Electro-Optical Interfacial Effects on a Graphene/π-Conjugated Organic Semiconductor Hybrid System. *Beilstein Journal of Nanotechnology* **9**, 963–974 (2018).
